# Ionic Liquid-Enhanced Interfaces to Boost Reactive CO_2_ Capture

**DOI:** 10.1021/acs.jpcb.5c07692

**Published:** 2026-02-19

**Authors:** Amey S. Thorat, Rohan Sartape, Rohit Chauhan, Rashmi Mishra, Meenesh R. Singh, Jindal K. Shah

**Affiliations:** † School of Chemical Engineering, 7618 Oklahoma State University, Stillwater, Oklahoma 74078, United States; ‡ Department of Chemical Engineering, 14681 University of Illinois Chicago, Chicago, Illinois 60608, United States

## Abstract

The addition of ionic liquids (ILs) to a mixture containing a molecular solvent and other ionic species can induce the heterogeneous redistribution of cations and anions at the gas–liquid interface. This nonuniform redistribution of cations and anions driven by the differences in the solvophilicity of ions can improve the thermophysical and interfacial properties of such mixtures, creating a local chemical environment that is conducive to some reactions. In this work, ILs are added to a mixture of potassium hydroxide (KOH) and ethylene glycol (EG), used as a reactive absorbent and electrolyte in the migration-assisted moisture-gradient (MAMG) process for CO_2_ capture. Molecular dynamics (MD) simulations are employed to probe into the effects of complex ion–ion and ion–solvent interactions and to examine the chemical composition at the gas–liquid interface. A total of 12 systems are investigated using molecular simulations to identify trends in the performance of IL additives based on the choice of cation, anion, and IL concentration. The cation effects are studied using IL additives based on 1-ethyl-3-methylimidazolium ([EMIM]^+^) and 1-butyl-3-methylimidazolium ([BMIM]^+^), while the impact of anions is examined using additives based on dicyanamide [DCA]^−^, triflate [TfO]^−^, bistriflimide [NTf_2_]^−^, and hexafluorophosphate [PF_6_]^−^ anions, respectively. The influence of the IL concentration is also evaluated at molar concentrations between 1% and 4%. The simulation results indicate that the use of IL additives can affect the physical CO_2_ solubility, surface tension, and the localization of CO_2_ around the [OH]^−^ ions at the gas–liquid interface. It is also evident that the choice of cations, anions, and IL concentration determines the extent to which the IL additives impact the local physicochemical properties. Physical dissolution, diffusive transport, and interaction with [OH]^−^ are critical intermediate steps toward reactive CO_2_ capture using a liquid absorbent. Hence, the improvement in one or more of these properties, aided by IL additives, is expected to improve the overall CO_2_ capture performance. Experiments reaffirmed the impact of IL additives on CO_2_ capture performance and the sensitivity to the choice of the cation, anion, and concentration of the IL additive.

## Graphical abstract

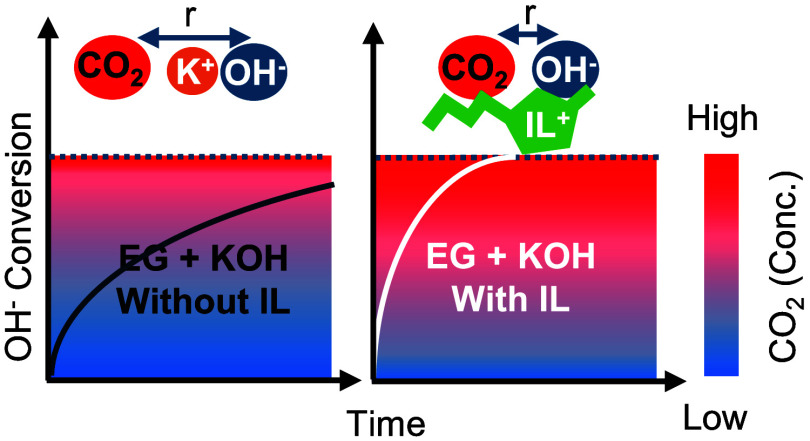

## Introduction

Global warming and climate change have necessitated the development of energy-efficient processes for the capture of atmospheric CO_2_. Many physical and chemical processes that leverage materials with strong physical affinity or chemical reactivity toward CO_2_ have evolved.^1–5^ However, purely physical processes are materials-intensive due to the limited CO_2_ loading capacity of the absorbents,^6,7^ while chemical processes are energy-intensive due to the need for reactant regeneration.^8,9^ This makes such processes inefficient to implement on a large scale. Electrochemical processes can offer energy-efficient alternatives by leveraging both the physical solubility and the chemical reactivity of an electrolyte. Among such processes is the migration-assisted moisture-gradient (MAMG) process^10^ that uses a nonaqueous mixture of EG and KOH as a reactive solvent for CO_2_ capture. The MAMG process captures CO_2_ through the reaction of CO_2_ with [OH]^−^ to form bicarbonate ([HCO_3_]^−^) ions in a nonaqueous environment. The [HCO_3_]^−^ ions migrate across an anion exchange membrane to the aqueous side and decompose in the presence of moisture to release the CO_2_. The performance of the MAMG process may be improved through fine-tuning of the thermodynamic, electrodynamic, and transport properties of the electrolyte by using compatible additives or cosolvents.^11^ ILs are chemically, thermally, and electrochemically stable and are compatible with a large number of molecular solvents and ionic species.^12^ Additionally, they have high ionic conductivity,^13,14^ and high molar CO_2_ solubility.^15–17^ These properties make ILs excellent candidates for incorporation into a CO_2_ capture electrolyte.^18^ However, the presence of polar and nonpolar moieties on the IL cations and anions induces complex ion–ion and ion–solvent interactions that can affect the local thermophysical and transport properties.^19–21^ These effects can be best understood by examining the systems at the molecular level, thereby motivating an *in silico* investigation as the first step toward the use of IL additives for improving the performance of the MAMG process.

Reactive CO_2_ capture using liquid reactants is a complex, multistep process that begins with the dissolution of CO_2_ from the gas phase into the liquid, followed by its diffusion within the bulk liquid, and finally the chemical reaction. The solvent regeneration step involves cleaving bonds between CO_2_ and the reactant. Each step involves an energy penalty associated with overcoming the thermodynamic and transport resistances. Nemukhin et al.^22^ investigated in detail the evolution of the solvation environment, and consequently the origin of the potential barrier for the reaction of CO_2_ and [OH]^−^ in an aqueous environment using cluster solvation and continuum models. They report that the solvation environment, molecular geometry, and partial atomistic charges on CO_2_ and [OH]^−^ evolve rapidly as these molecules approach closer than 4.0 Å to each other. They also predicted a reaction energy barrier as molecules approach each other. Various strategies have evolved to mitigate these barriers, such as through the use of cosolvents, surfactants, and catalysts. For example, the use of solvents and additives with high CO_2_ solubility can improve the physical solubility of CO_2_,^23–25^ while the use of low viscosity cosolvent improves the transport properties of the mixture.^26–28^ Surfactants can disrupt hydrogen bonding networks at the gas–liquid interface, thereby creating gateways or favorable regions for CO_2_ to enter the liquid at the interface.^29–32^ In their work, Pichetwanit et al.^33^ have demonstrated that cationic surfactants such as dodecyltrimethylammonium bromide (DTAB) improve CO_2_ loading capacity and CO_2_ loading rates, while nonionic surfactants reduce the surface tension of monoethanolamine (MEA) solutions. Bryant et al.^34^ have reported improved CO_2_ capture performance due to surfactants in MEA solutions using packed and bubble columns. The use of catalysts can offer energy-efficient reaction pathways^35^ or efficient solvent regeneration.^36^ The aforementioned strategies alter the CO_2_-solvent or CO_2_-reactant interactions,^37^ and have been employed independently or in tandem to improve the overall CO_2_ capture efficiency. An extremely large chemical space and the versatile chemistry of ILs allow us to incorporate multiple such strategies into a single additive by engineering the molecular structures of the cations and anions. Molecular simulations can unravel the specific mechanisms^38^ at the molecular level that yield these effects, and hence, an *in silico* investigation emerges as a first step to examine the effects of IL additives on the reaction mixture.

Neat ILs have continued to be explored as solvents for CO_2_ capture over the last two decades.^39–42^ These include ILs for both physical absorption,^17^ as well as reactive CO_2_ capture.^43–46^ However, strong ionic interactions and bulky chemical structures often impart high viscosity to pure ILs.^47–50^ Also, multistep synthesis,^51,52^ potential toxicity,^53,54^ and high production costs pose challenges toward the large-scale application of some ILs. These challenges can be overcome through the use of binary mixtures of ILs and molecular solvents. For example, it is well-known that IL-molecular solvent mixtures, at sufficient IL concentrations, yield higher ionic conductivity than neat ILs due to better solvation and ion dynamics.^50,55–58^ The use of ILs in catalysis is also well documented, in the role of a catalyst, cocatalyst, or catalytic solvent.^59–62^ Although ILs have been investigated for their ability to capture CO_2_ in bulk and confinement, their application as additives remains largely unexplored. These factors drive our investigation to gauge the benefits of incorporating IL additives rather than ILs as bulk or in confinement toward reactive CO_2_ capture using the MAMG process.

This work examines the effects of IL additives on surface tension, physical CO_2_ solubility, and local chemical environment at the gas–liquid interface of a nonaqueous reactive CO_2_ capture mixture containing KOH and EG. The influence of the choice of IL cations was investigated using additives based on 1-ethyl-3-methylimidazolium [EMIM]^+^ and 1-butyl-3-methylimidazolium [BMIM]^+^. The impact of anion solvophobicity was analyzed by using IL additives containing dicyanamide [DCA]^−^, triflate [TfO]^−^, bistriflimide [NTf_2_]^−^, and hexafluorophosphate [PF_6_]^−^. The chemical structures of the IL cations and anions in this study are presented in Figure 1. The effect of the IL concentration was probed at molar IL concentrations between 1% and 4%. Molecular dynamics (MD) simulations were performed to obtain CO_2_ Henry’s constants, surface tension, and local chemical composition at the gas–liquid interface. Due to the limitations of using a nonreactive force field, trends in reaction kinetics were indirectly inferred using molecular configurations where the distance between [OH]^−^ and CO_2_ was less than 3.5 Å. The relative abundance and location of such configurations across systems containing different IL additives suggested trends in the reactivity. While MD simulations offered a molecular-level insight into the prereaction dynamics of CO_2_ and various ionic species, experiments helped assess the impact of IL additives on CO_2_ capture rates and their sensitivity to the choice of IL cation, anion, and concentration.

**Figure 1:**
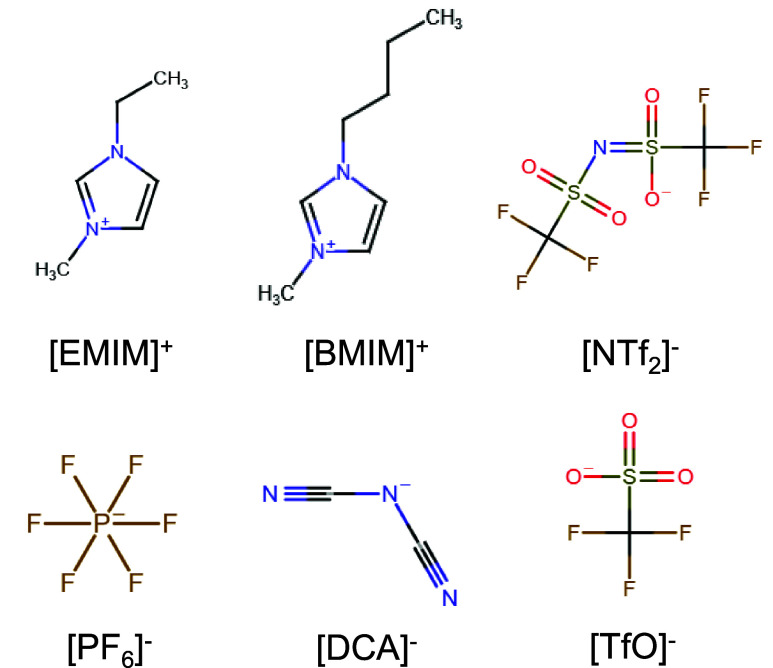
Molecular structures of IL cations and anions used in this work.

## Methods

### Simulation

#### Force Fields

MD simulations were performed using GROMACS 2018.^63–70^ Imidazolium ILs were represented using the virtual site force field for ionic liquids (VSIL) developed by Doherty et al.,^71^ while EG was represented using the OPLS-DES force field,^72,73^ both derived from the nonpolarizable OPLS all-atom force field.^74^ The force field developed by Jensen and Jorgensen^75^ was used to represent the [K]^+^ ion following an earlier work,^19^ while the force field developed by Habibi et al.^76^ was used to represent [OH]^−^. The force field parameters for CO_2_ were taken from the work of Shi and Maginn.^77^ The total energy is estimated based on the bonded and nonbonded interactions among the atoms. The intramolecular bonded interactions are resolved into harmonic stretching, bond angles, and dihedrals, while Coulombic and Lenard-Jones (LJ) terms contribute toward the nonbonded interactions. Geometric combination rules were used for the estimation of LJ parameters for unlike interactions. All 1–4 nonbonded interactions were scaled using a factor of 0.5. A cutoff distance of 16 Å was used to apply the tail corrections for electrostatic and LJ interactions. The PME method was used to compute the long-range electrostatic energy.

#### Estimation of CO_2_ Solubility

The effect of IL additives on the physical gas solubility was evaluated based on Henry’s constant (*k*_H_) evaluated at infinite dilution of CO_2_ using eq 1

1 $$k_{H}=\rho RT\;\exp\frac{\mu^{\mathrm{ex}}}{k_{B}T}$$

where ρ represents the molar density of the solvent liquid, *R* is the universal gas constant, *T* is the temperature, μ^ex^ is the excess chemical potential, and *k*_B_ is Boltzmann’s constant. The excess chemical potential μ^ex^ was estimated using the Bennett’s Acceptance Ratio (BAR) approach.^78^ Here, the strength of interaction of CO_2_ with the solvent is varied by using a coupling parameter λ. The solute–solvent interactions were sequentially turned off by scaling the λ parameter from one to zero in 11 steps each for Coulombic and LJ interactions, respectively. Thus, 22 separate MD simulations were performed to obtain the *k*_H_ value for CO_2_ in a mixture with an IL additive. The process was repeated for IL additives of interest to obtain trends in the physical solubility. Each simulation was performed using a system of 400 molecules of EG, 26 ions each of [K]^+^ and [OH]^−^, 5 ion-pairs of the IL additive, and 1 molecule of CO_2_. The molecules were randomly packed in a cubic box as a starting configuration using Packmol,^79^ followed by an energy minimization step to avoid any high-energy configurations or overlapping atoms. This was followed by a high-temperature annealing step, and equilibration using the canonical *NVT* ensemble (10 ns), and *NPT* ensemble (10 ns), a preproduction run (10 ns), and a production run (20 ns) using the *NPT* ensemble. The temperature was maintained at 298 K using Langevin dynamics, and pressure was maintained using Parrinello–Rahman^80^ pressure coupling. Henry’s constant was first evaluated for a system without an IL additive as a reference, while the effect of the anions was estimated on separate systems containing [BMIM]­[DCA], [BMIM]­[PF_6_], [BMIM]­[TfO], and [BMIM]­[NTf_2_] as additives.

#### Simulation of Bulk Liquid

Systems containing EG, KOH, IL, and CO_2_ were simulated to identify changes in bulk liquid properties due to the addition of the ILs. In the first step, bulk simulations were performed using 3400 EG molecules, 225 ion-pairs of [K]^+^ and [OH]^−^, 40 ion-pairs of the IL additive, and 40 molecules of CO_2_. In the first step, all species were packed into cubic boxes using Packmol^79^ to obtain an initial configuration. Energy minimization and high-temperature annealing (2 ns) were performed to eliminate high-energy configurations or local minima. The system was equilibrated using the *NVT* and *NPT* ensembles for 10 ns each. Equilibration was followed by a preproduction of 10 ns and a production run of 20 ns using the *NPT* ensemble. System temperature was maintained at 298 K using the Nosé-Hoover thermostat^81^ with a time constant of 0.4 ps, and pressure was maintained at 1.0 bar using the Parrinello–Rahman pressure coupling^80^ with a time constant of 2.0 ps. In the second step, the density obtained by using the *NPT* production run of the first step was used as a reference to generate an initial configuration. The system was then subjected to energy minimization, high-temperature annealing (2 ns), equilibration (10 ns), and production run (100 ns) by using the *NVT* ensemble. System temperature was maintained at 298 K using the Nosé-Hoover thermostat^81^ with a time constant of 0.4 ps.

#### Simulation of the Gas–Liquid Interface with CO_2_

A liquid slab was simulated to examine the distribution of the ions at the interface. Initial configurations of slabs containing 3400 molecules of EG, 225 ion-pairs of [K]^+^ and OH^–^, 40 ion-pairs of IL, and 100 molecules of CO_2_ were prepared by packing the liquid in boxes of dimensions 72 Å × 72 Å × 216 Å in the *x*, *y*, and *z* directions, respectively. The liquid was packed in the center along the *z*-direction with vacuum regions equal to one box length (about 72 Å) on both sides of the liquid slab. The slabs were subjected to energy minimization and followed by equilibration (20 ns), preproduction (20 ns), and production run (200 ns) using the canonical *NVT* ensemble. System temperature was maintained at 298 K using a velocity-rescale thermostat^82^ with a time constant of 1.0 ps during equilibration, and using the Nosé-Hoover thermostat^81^ with a time constant of 0.4 ps during production. The slab simulation results were used to analyze the surface tension and distribution of ionic species at the interface. The systems with CO_2_ were simulated with 100 CO_2_ molecules initially placed inside the liquid slab.

### Experiment

KOH (99.99% purity) pellets and KHCO_3_ and EG (99% purity) were obtained from Sigma-Aldrich. Ionic liquids were obtained from IoLiTec GmbH and used without further purification. EG and ILs were both dried in a vacuum oven at 60 °C. CO_2_ (99.99%) and N_2_ (99.999%) tanks were obtained from Praxair. The reactive absorbents were prepared by dissolving an appropriate amount of KOH in EG to obtain 0.1 M KOH. IL additives were added appropriately to measure 5%, 10%, and 20% by volume, which correspond to approximately 1%, 2%, and 4% molar concentrations.

The reaction of CO_2_ and [OH]^−^ proceeds through the formation of the [HCO_3_]^−^ ion. The progress of the reaction was monitored using the ionic conductivity of the reaction mixture via an automated screening platform. Additional benchmarking of the automated screening platform was carried out using a conductivity meter (Orion Star A212 Conductivity Benchtop Meter, Thermo Scientific, USA) and a conductivity probe (Orion 013005MD, Thermo Scientific, USA). To achieve this, first, a calibration curve was obtained for reference systems by creating solutions with known concentrations of EG, KOH, KHCO_3_, and the IL additive. The molal concentration of KOH was decreased from 0.1 to 0 M, while the concentration of KHCO_3_ was increased from 0 to 0.1 M to mimic the progress of the reaction. The temperature was maintained at 25 °C. The reaction was carried out by bubbling 10% CO_2_ (balance N_2_) at the rate of 10 mL/min through 400 μL of well-mixed reaction mixture containing EG, KOH, and IL additives. Ionic conductivity of the reaction mixture was recorded periodically. The [OH]^−^ conversion was estimated by comparing the ionic conductivity values with the corresponding values in the calibration curve of each system.

## Results and Discussion

### Distribution of IL Cations and Anions

Figure 2a depicts a simulation snapshot of a liquid slab containing the absorbent mixture containing EG, KOH, and the IL additive. CO_2_ molecules dissolve in and effuse out of the liquid slab throughout the simulation. To better assess the distribution of the chemical species in the direction normal to the gas–liquid interface, we calculated their distance from the midpoint of the liquid slab along the *z*-direction at each snapshot. These distances are termed the L-coordinate. Thus, *L* = 0 indicates the midpoint of the slab, i.e., the bulk liquid. The location of the gas–liquid interface is system-specific and generally occurs between L-coordinates of 35 to 42 Å. System equilibration was assessed by comparing the radial distribution functions (RDFs) around the center of mass (COM) of IL anions from 100 to 130 ns and 170–200 ns of the production run trajectory (refer to Figures S2 and S3). Anions were selected as a reference since they exhibit differences in their solvophilicity and CO_2_ affinity. The nearly identical RDFs across the two time frames suggest that the overall structure and distribution are maintained through the trajectory. Minor differences are expected from fluctuations in the number of dissolved CO_2_ molecules.

**Figure 2:**
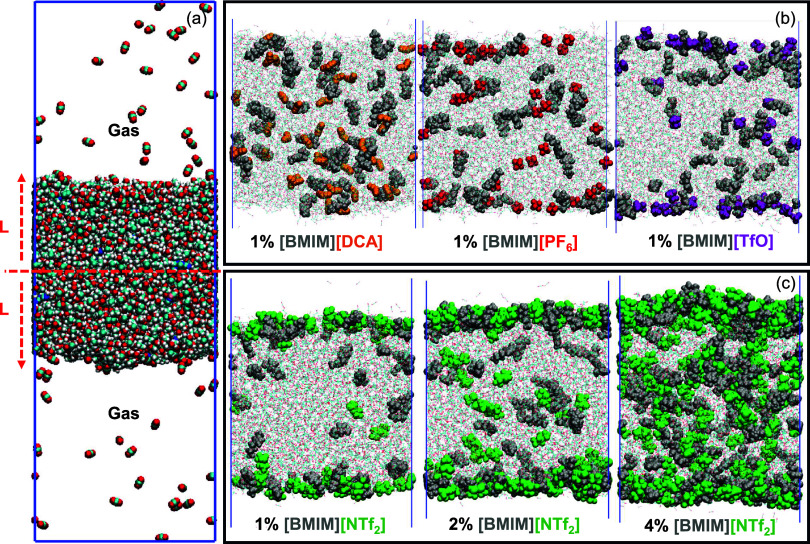
(a) Simulation box containing the reactive mixture (EG-KOH + IL additive) and CO_2_ gas. The distance *L* is measured from the center of a slab. Snapshots highlighting the position of [BMIM]^+^ and anions in different systems with (b) 1% [BMIM]­[X] additives and (c) different concentrations of [BMIM]­[NTf_2_].

Figure 2b illustrates the differences in the distribution of [BMIM]^+^ cations and corresponding anions at a 1% IL additive concentration. Figure 2c compares the ion distribution at 1%, 2%, and 4% concentrations of [BMIM]­[NTf_2_]. It can be observed that the distribution of the ions is influenced strongly by the choice of the anion. Anions such as [NTf_2_]^−^ tend to concentrate at the interface, while [DCA]^−^ and [PF_6_]^−^ are distributed throughout the liquid. The heterogeneity in the anion distribution induces corresponding nonuniformity in the distribution of the [BMIM]^+^ ions. At 1% IL, the cations are concentrated near the interface. However, the cations and anions begin to penetrate into the bulk liquid with an increase in the IL concentration. The trends in the distribution of COMs of ions in systems with [EMIM]^+^ based ILs (refer to Figure S1) were found to be quite similar to the [BMIM]^+^ systems. However, the smaller [EMIM]^+^ ions with shorter alkyl tails are more solvophilic than [BMIM]^+^, and have a higher number density in the bulk liquid than their [BMIM]^+^ counterparts.

Figure 3a represents the number density for the [BMIM]^+^ COMs along the L-coordinate in systems at 1% additive concentration. All distributions were averaged over 10,000 snapshots taken every 10 ps between 100 and 200 ns of the production run. The region to the left of the peak represents the liquid, while the region to its right indicates the gas phase. The corresponding distributions of the [EMIM]^+^ systems are available in Figure S4. Figure 3a indicates that the distribution of the cation is sensitive to the choice of the anion, with the highest number density occurring at the interface when paired with a solvophobic anion such as [NTf_2_]^−^. Shorter peaks at the interface and increased presence in the bulk are observed when [BMIM]^+^ is paired with solvophilic anions such as [PF_6_]^−^ and [DCA]^−^. Figure 3b illustrates the corresponding distributions of COMs for various anions. The peak number density occurs at the interface. The peak height indicates the relative solvophobicity, with [DCA]^−^ as the most solvophilic, and [NTf_2_]^−^ as the most solvophobic anion. Figure 3c,d depicts the number density of [BMIM]^+^ and [NTf_2_]^−^ COMs as a function of the [BMIM]­[NTf_2_] concentration. The increase in the IL concentration is reflected in the increased number density within the bulk and at the interface alike. The peaks occur at higher L values with the addition of the IL additive due to the increase in the volume of the liquid. Similar trends were observed in the distribution of [EMIM]^+^ systems, with less prominent peaks at the interface than the corresponding [BMIM]^+^ systems (see Figure S4).

**Figure 3:**
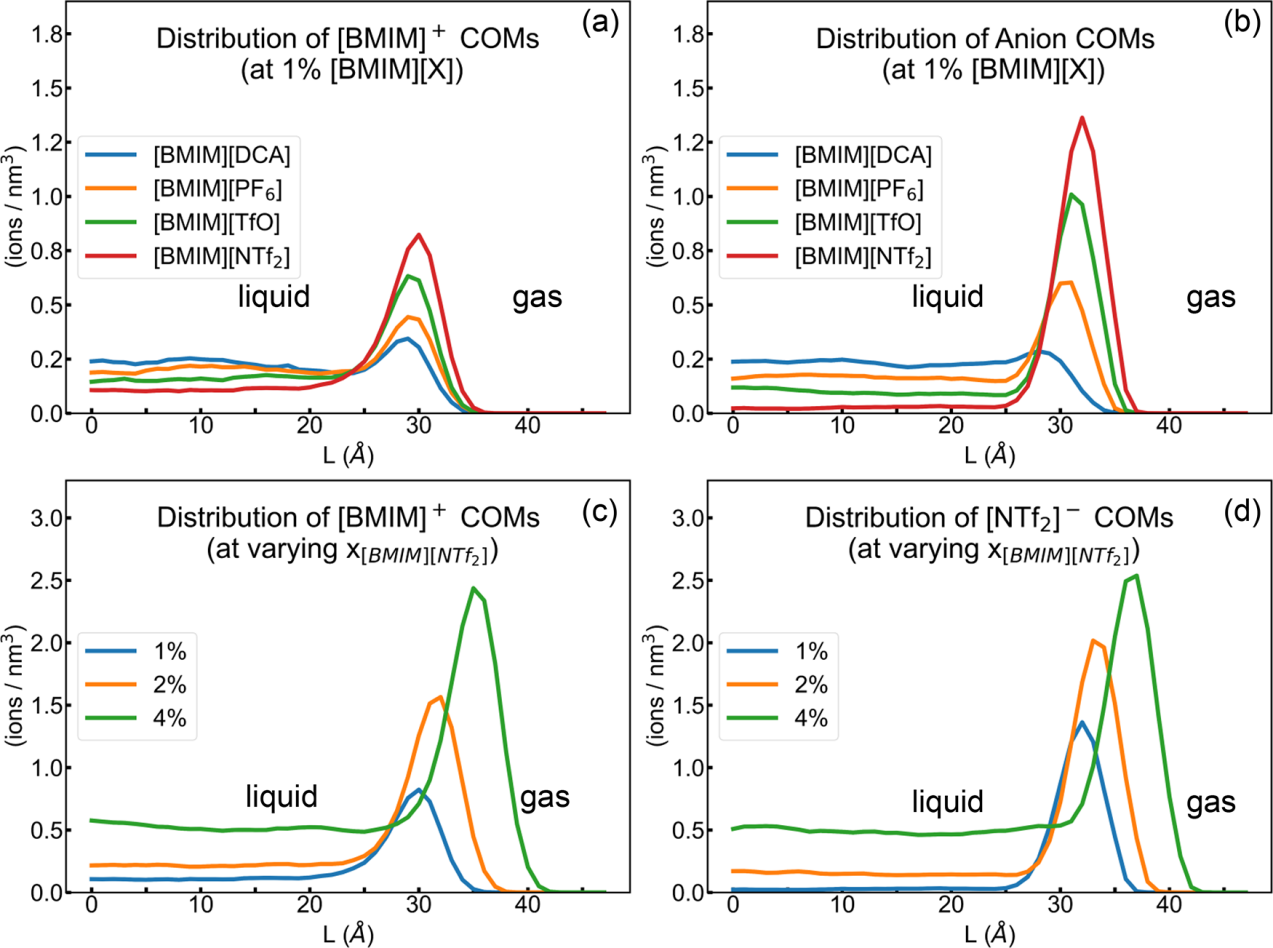
Distribution of COMs of IL cations and anions along the L-coordinate (a) [BMIM]^+^ in 1% [BMIM]^+^ ILs. (b) Anions in 1% [BMIM]^+^ ILs. (c) [BMIM]^+^ in [BMIM]­[NTf_2_]. (d) [NTf_2_]^−^ in [BMIM]­[NTf_2_] systems.

We also examined the effect of force fields on the number density distribution of [EMIM]^+^ and [NTf_2_]^−^ in systems containing 1% [EMIM]­[NTf_2_] additive using the VSIL, 0.8*2009IL,^83^ and 2009IL^84^ force fields (refer to Figure S8). Despite some differences in the number density distribution for COMs of these ions, a heterogeneous distribution was observed across all three force fields, with the anions and cations concentrating near the interface. The 2009IL force field yielded the lowest ion density near the interface due to a higher magnitude of charges ± 1 *e* ^–^ on the IL cation and anion compared to the other two force fields with ± 0.8 *e* ^–^ charge.

Thus, it is evident that the distribution of IL cations and anions is in homogeneous and influenced by the choice of the cations, anions, and IL concentration when added to the EG-KOH mixture. The surface accumulation of solvophobic ions is expected to influence the interfacial properties of the mixture, such as its surface tension.

### Surface Tension

Figure 4a represents the surface tension calculated at 1% IL concentration as a function of the IL anions, while Figure 4b depicts the surface tension of the mixtures containing [NTf_2_]^−^ ILs at various concentrations. Surface tension was calculated using liquid slab simulations. Surface tension tends to be lower in additives containing solvophobic anions such as [NTf_2_]^−^ and [TfO]^−^. Among cations, additives with longer solvophobic alkyl chains, such as [BMIM]­[TfO] and [BMIM]­[NTf_2_], yield lower surface tension compared to [EMIM]^+^ based additives. For comparison, the surface tension for pure EG is about 48.5 dyn/cm,^85^ [EMIM]­[NTf_2_] is 36.5 dyn/cm,^86^ and [BMIM]­[NTf_2_] is 32 dyn/cm.^87^ With suitable IL additives, the surface tension of the mixture is lower than that of pure EG but remains higher than pure IL. For example, with 4% [BMIM]­[NTf_2_] additive, the surface tension of the mixture drops to 40 dyn/cm, or approximately by 20%. Lowering the surface tension reduces the gas bubble diameter, increases the surface area, and can help improve the mass transfer of CO_2_.

**Figure 4:**
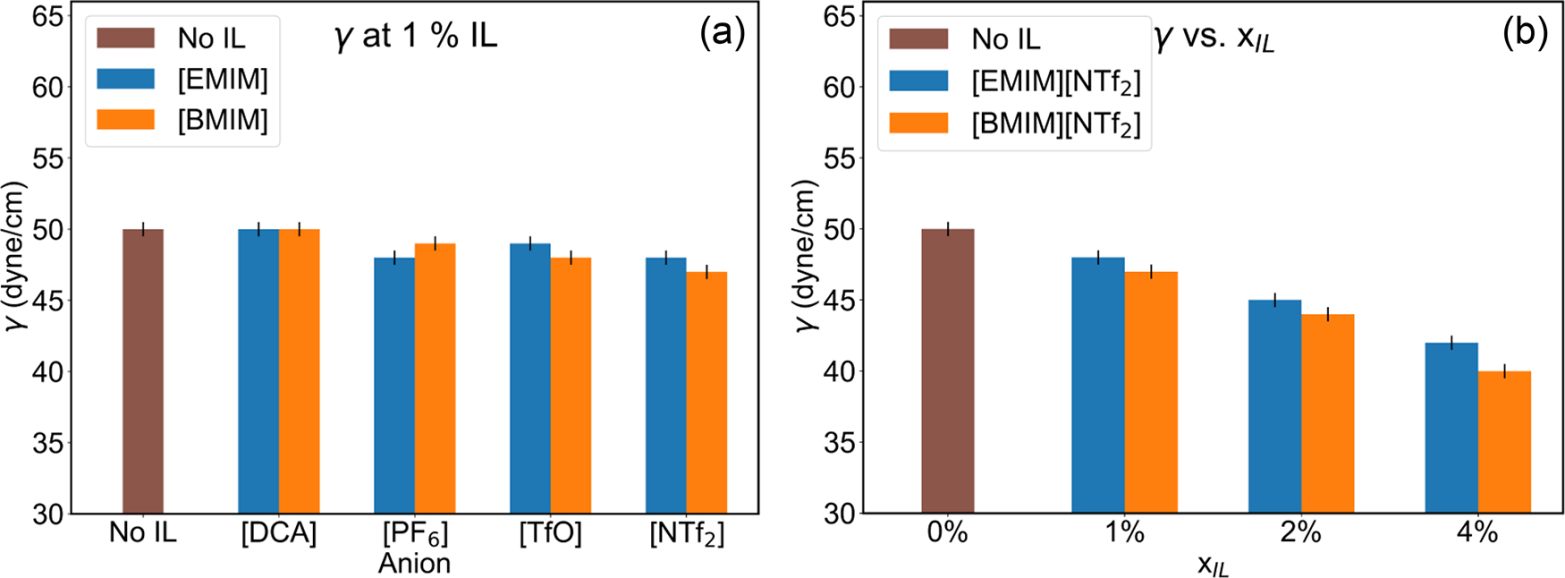
Surface tension (γ) of the reaction mixture (a) at 1% IL additive concentration (b) varying *x*_IL_ in [NTf_2_]^−^ ILs.

### Physical CO_2_ Solubility

ILs are known for their high molar CO_2_ solubility. Although the MAMG process is a reactive electrochemical process, [OH]^−^ ions remain within the electrolyte and the dissolution of CO_2_ from the gas phase into the liquid is a prerequisite to its reaction with [OH]^−^. In fact, in a situation where the reaction occurs very quickly, the dissolution of CO_2_ becomes a rate-determining step, and the process can become mass-transfer limited. Hence, an improvement in physical CO_2_ solubility is equally important in the context of the MAMG process.

Table 1 provides the experimental Henry’s constant values for CO_2_ ( $k_{H_{CO_{2}}}$ ) reported for pure compounds. When expressed in pressure units, the CO_2_ solubility is inversely related to the *k*_HCO_2__ value. Thus, lower values of *k*_HCO_2__ indicate higher CO_2_ solubility. Molar CO_2_ solubility in neat ILs is approximately an order of magnitude higher than that in neat EG. Further, among pure ILs, it is known that *k*_HCO_2__ values are sensitive to the choice of anions,^94,97^ for example, *k*_HCO_2__ for [EMIM]­[DCA] is nearly twice that for [EMIM]­[NTf_2_]. Among ILs containing the same anion, *k*_HCO_2__ values decrease with the longer alkyl chains on the cation. Thus, CO_2_ solubility follows the order [EMIM]­[NTf_2_] < [BMIM]­[NTf_2_]. Substantial variation in the reported solubility values can be observed, which is attributed to the sensitivity of the CO_2_ solubility to moisture content and impurities.

**Table 1:** CO_2_ Solubility in Pure Compounds

	*k*_HCO_2__ (bar)
EG	454,^88^ 262 ^89^
[EMIM][DCA]	82 ^90^
[EMIM][NTf_2_]	36,^15^ 40,^91^ 42 ^92^
[BMIM][PF_6_]	52,^93^ 65,^90^ 55 ^94^
[BMIM ][NTf_2_]	32,^95^ 37,^96^ 28 ^94^

Figure 5a denotes the *k*_HCO_2__ values calculated from MD simulations using a free energy-based approach for reaction mixtures containing EG, KOH, and [BMIM]^+^-based additives. It is evident that even with 1% IL, the *k*_HCO_2__ values are lowered by approximately 20% compared with the EG-KOH mixture without the IL additive. For mixtures, the *k*_HCO_2__ values calculated from MD simulations were observed to be slightly lower than the values predicted using eq 2.

2 $$\frac{1}{k_{H_{CO_{2}\left(\mathrm{EG}+\mathrm{KOH}+\mathrm{IL}\right)}}}=\frac{x_{\mathrm{IL}}}{k_{H_{CO_{2}\left(\mathrm{IL}\right)}}}+\frac{\left(1-x_{\mathrm{IL}}\right)}{k_{H_{CO_{2}\left(\mathrm{EG}+\mathrm{KOH}\right)}}}$$

where *k*_HCO_2__(EG+KOH)__, *k*_HCO_2__(IL)__, and *k*_HCO_2__(EG+KOH+IL)__ represent the CO_2_ Henry’s constants in EG + KOH, pure IL, and EG + KOH with IL additive, respectively. *x*_IL_ denotes the mole fraction of the IL additive. eq 2 enables us to predict *k*_HCO_2_(EG+KOH+IL)_ at different IL concentrations. The deviation between the predicted and calculated values may be attributed to the force field effects.^98^ It is observed that the *k*_HCO_2__ values drop by about 40% at a 5% molar IL concentration. However, since the IL molecules are large with strong interactions, an increase in the IL concentration leads to an increase in the solution viscosity, translating into an increase in the material and operating costs. Hence, determining the optimum IL concentration is critical to achieving the most beneficial impacts on the overall CO_2_ capture process.

**Figure 5:**
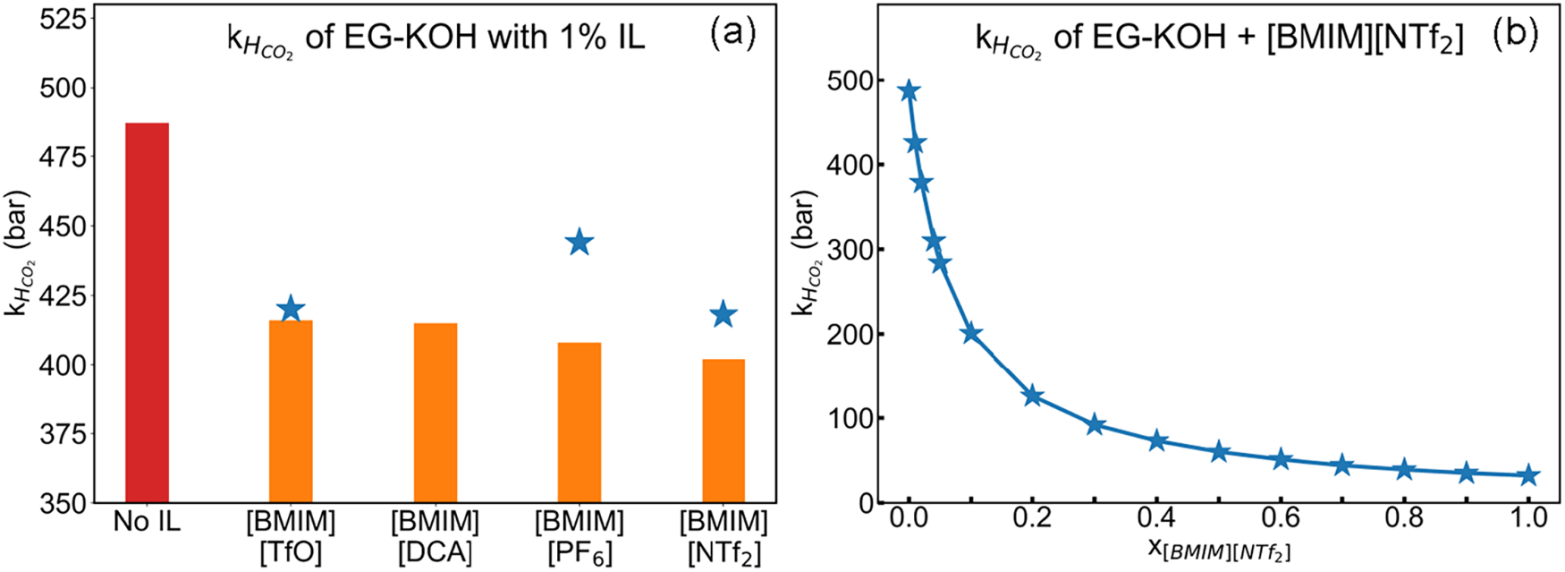
(a) Comparison of CO_2_ Henry’s constant values calculated using MD simulations for EG+KOH mixtures containing [BMIM]­[X] additives with varying anions (bars) with values predicted using eq 2 (blue markers). (b) CO_2_ Henry’s constant values predicted using eq 2 at varying concentrations of [BMIM]­[NTf_2_].

### Distribution of Dissolved CO_2_

Figure 6a depicts the number of CO_2_ molecules dissolved in the liquid from a total of 100 CO_2_ molecules evolving across 2000 snapshots taken at every 50 ps from 100 to 200 ns of the production run trajectory. It is evident that the addition of [BMIM]­[NTf_2_] to the mixture yields a higher number of dissolved CO_2_ molecules. The number of CO_2_ molecules also increases with the concentration of [BMIM]­[NTf_2_], in accordance with the enhanced solubility of CO_2_ as depicted in Figure 5b. Fluctuations in the number of CO_2_ molecules in the liquid indicate frequent exchanges of gas molecules across the interface.

**Figure 6:**
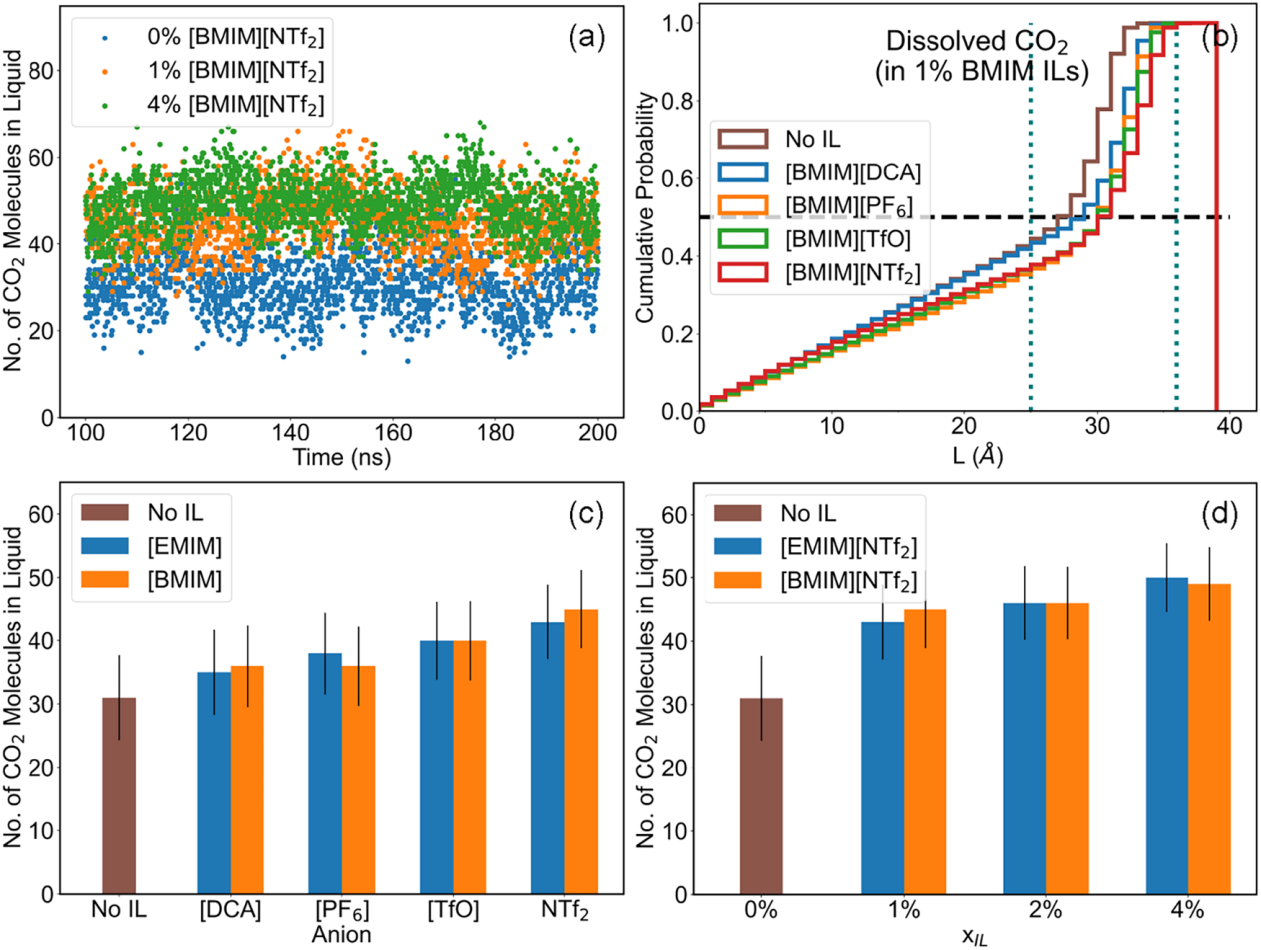
(a) Number of dissolved CO_2_ molecules (out of 100) in systems containing [BMIM]­[NTf_2_] at various IL concentrations. (b) Cumulative distribution of CO_2_ along L-coordinate in 1% [BMIM]^+^ ILs Black dashed line indicates median population of dissolved CO_2_, blue dotted lines indicate region up to 1 nm from the interface. (c) Average number of dissolved CO_2_ molecules at 1% IL concentration. (d) Average number of dissolved CO_2_ in [NTf_2_]^−^ based ILs at various concentrations.

Figure 6b illustrates the cumulative distribution of CO_2_ molecules along the L-coordinate. Approximately 50% of the dissolved CO_2_ population is located within 1 nm of the gas–liquid interface. These values are likely to be influenced by the surface area to volume ratio of the system, but they do suggest that the interface is generally rich in CO_2_ across all systems, with or without the addition of ILs. Simulation data indicate that the accumulation of CO_2_ near the interface is prominent in the mixtures with the IL additive.

Figure 6c presents the trend in the average number of CO_2_ molecules in the liquid across systems with 1% IL additives. In general, the addition of ILs increases the CO_2_ solubility, with [TfO]^−^ and [NTf_2_]^−^ based ILs yielding approximately 30% more CO_2_ molecules in the liquid. Among IL additives containing the same cation, the trend in the number of CO_2_ molecules in the liquid closely follows the trend in the physical solubility of CO_2_ in neat ILs, as shown in Table 1. Thus, [BMIM]­[NTf_2_] and [EMIM]­[NTf_2_] absorb more CO_2_ than [BMIM]­[DCA] and [EMIM]­[DCA], respectively.

Figure 6d helps to analyze the trend in the number of CO_2_ molecules absorbed in liquid as a function of IL concentration. CO_2_ in mixtures with [EMIM]­[NTf_2_] or [BMIM]­[NTf_2_] additives increases with the IL concentration, with 4% [BMIM]­[NTf_2_] absorbing nearly 50% more CO_2_ molecules compared to a neat EG-KOH system. These observations are in agreement with the behavior expected from eq 2.

We caution readers that the estimation of exact CO_2_ solubility was not the primary aim of this exercise. Since a fixed number of CO_2_ molecules was used for simulations in the *NVT* ensemble, the fluctuations in the number of dissolved CO_2_ molecules introduce corresponding fluctuations in the net pressure of the system. Therefore, such simulations do not accurately represent real-world systems. Here, we limit the use of this approach to the qualitative comparison of dissolution and effusion behavior of CO_2_ across systems containing different IL additives. Nonetheless, it is evident that incorporation of IL additives into the EG-KOH electrolyte improves the physical CO_2_ solubility, thereby facilitating better CO_2_-[OH]^−^ interactions as discussed next.

### Localization of [OH]^−^ around CO_2_

Figure 7a represents the radial distribution function (RDF) of [OH]^−^ around CO_2_ at a 1% [BMIM]^+^ IL additive concentration, calculated from the bulk liquid simulations. The RDFs indicate a smaller peak at a distance of about 4 Å, and a taller, broader peak at 7 Å. This distribution of CO_2_ is expected due to the strong Lewis acid–base interactions between the two species. Unlike the bulkier IL cations, the small size and nearly five times higher concentration of [K]^+^ ions in the electrolyte cause them to localize densely around [OH]^−^. This is evident from the taller peaks associated with [K]^+^(refer to Figure S6c), which arise from a strong interaction between [K]^+^ and [OH]^−^ within the first solvation shell. In contrast, CO_2_, being an electrically neutral molecule, is typically located farther away from the ions and needs to overcome the [K]^+^ ions to interact with the [OH]^−^.

**Figure 7:**
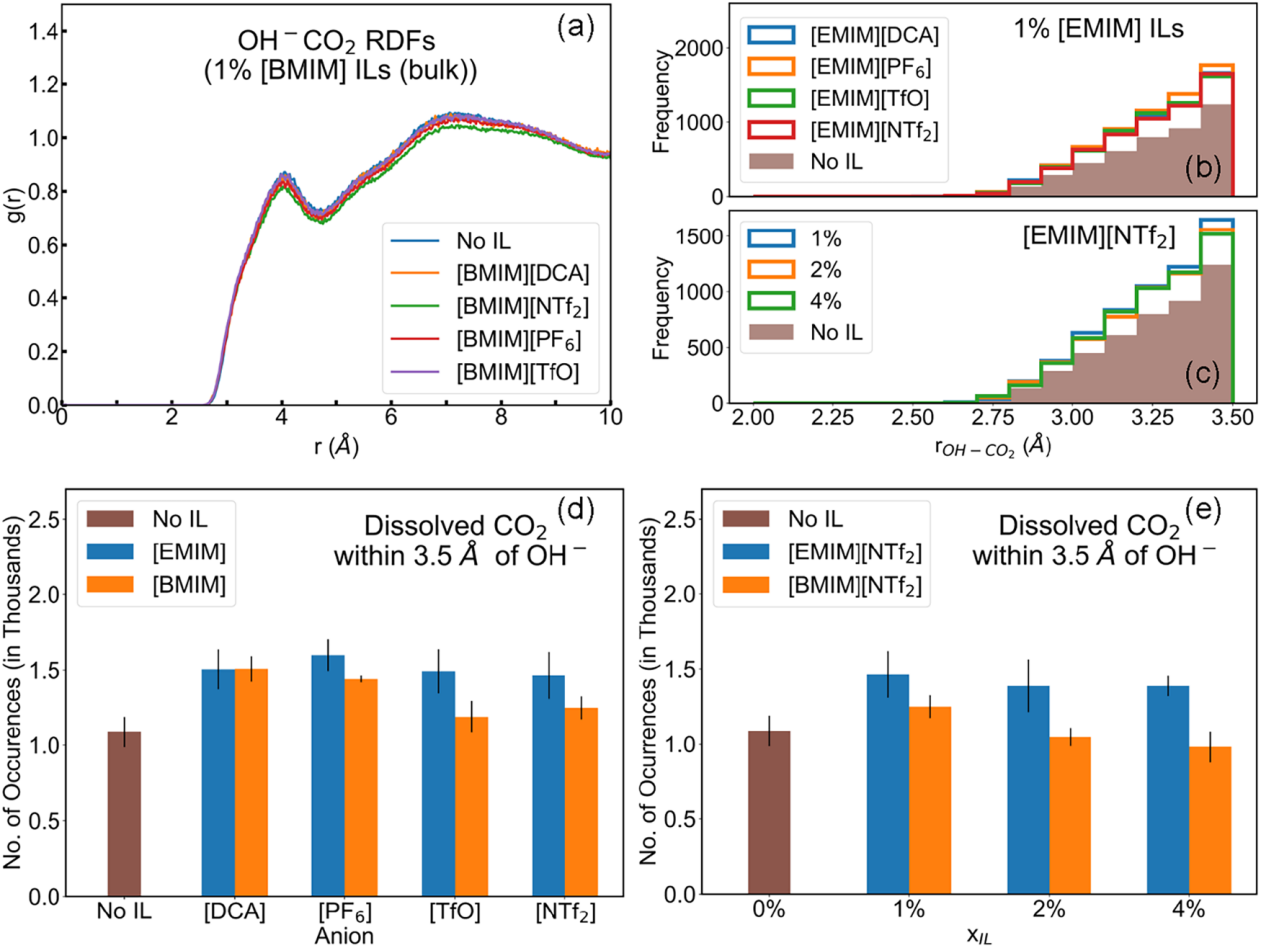
(a) RDF for COMs of [OH]^−^ and CO_2_ with 1% [BMIM]^+^ IL additives. Distribution of CO_2_-[OH]^−^ distance up to 3.5 Å with (b) 1% [EMIM] IL additives and (c) varying *x*_[EMIM][NTf_2_]_. Number of occurrences of CO_2_ within 3.5 Å of [OH]^−^ with (d) 1% IL additives and (e) varying x_IL_ in [NTf_2_]^−^ based systems across 2500 snapshots.

RDFs represent a picture of the relative distribution of the target species around a reference averaged over time and the number of particles. Hence, they are less sensitive to resolving rare events such as the collisions of two species. Moreover, RDFs fail to capture the system geometry of a heterogeneous system, such as a liquid slab. Hence, we calculated the distance to the nearest [OH]^−^ ion for all dissolved CO_2_ molecules (*r*_[CO_2_][OH^–^]_) in each of the 10000 snapshots between 100 and 200 ns to sample the occurrences of configurations that can potentially lead to a reaction.

Nemukhin et al.^22^ have demonstrated that significant changes occur in the solvation environment, geometry, and atomistic charges of [OH]^−^ and CO_2_ molecules as they approach within 3.5 Å of each other in an aqueous environment. There is an energy penalty associated with the desolvation of the reactants, molecular bending, and reconfiguration of the chemical bonds during the reaction. This potential barrier limits the likelihood of an [OH]^−^-CO_2_ interaction leading to a reaction. The chances of a reaction can be increased by increasing the frequency of the collisions of the reactants, i.e., by bringing the reactants closer, more often; or by facilitating their interaction through the formation of a reaction intermediate, such as with the aid of a catalyst. While ILs are known to exhibit catalytic effects, it is beyond the scope of this work to probe into such activity. Here, we limit ourselves to examining the effects of IL additives on the physical interactions, specifically by comparing the number of occurrences where [OH]^−^ and CO_2_ get within 3.5 Å of each other, which would eventually facilitate both catalytic and noncatalytic effects to take place. Hence, the configurations where *r*_[CO_2_][OH^–^]_ < 3.5 Å become most critical for this work.

Figure 7b represents a histogram of the *r*_[CO_2_][OH^–^]_ distance shown up to 3.5 Å in systems containing 1% [EMIM]^+^ based additives. Dissolved CO_2_ was used as a reference while calculating the distance to the nearest [OH]^−^, to avoid counting any gas-phase CO_2_ molecules that may come within 3.5 Å of an [OH]^−^ ion but bounce off the interface. Figure 7c represents a similar histogram in the [EMIM]­[NTf_2_] systems at different concentrations. While it is difficult to resolve the cation or anion effects from the histogram of the [OH]^−^-CO_2_ distance, these histograms suggest that the addition of ILs increases the number of occurrences where *r*_[CO_2_][OH^–^]_ < 3.5 Å, thereby improving the localization of [OH]^−^ and CO_2_.

Figure 7d compares the number of occurrences of favorable configurations with *r*_[CO_2_][OH^–^]_ < 3.5 Å, sampled across 2500 snapshots in systems with 1% IL additive. It is evident that the presence of an IL increases the number of such occurrences. This increase in the frequency of interaction of CO_2_ and [OH]^−^ creates an opportunity for improving the CO_2_ capture rate.

Figure 7e illustrates the number of occurrences with *r*_[CO_2_][OH^–^]_ < 3.5 Å, sampled from 2500 snapshots in [NTf_2_]^−^ systems at different IL concentrations. We observe a slight decrease in the number of occurrences with an increasing concentration of [NTf_2_]^−^ ILs. This is due to the localization of dissolved CO_2_ around an increasing population of CO_2_-philic [NTf_2_]^−^ anions, reducing their presence around [OH]^−^. This is evident from the number density around CO_2_, which increases for [NTf_2_]^−^ and decreases for [OH]^−^ at higher IL concentrations (refer to Figure S7a,b). Similarly, an examination of the chemical environment around [OH]^−^ ions reveals that the number density of IL cations rapidly increases around 3.75 Å, significantly exceeding the number density of CO_2_ (refer to Figure S7c,d). Excess additive can hinder the [OH]^−^-CO_2_ interactions due to the localization of IL cations around [OH]^−^. Hence, the IL concentration is critical in determining the impact of the additive on the overall CO_2_ capture performance.

### Distribution of [OH]^−^ and CO_2_

Figure 8a illustrates the number density of the configurations where *r*_[CO_2_][OH^–^]_ < 3.5 Å along the L-coordinate in systems with 1% [EMIM]^+^ additives. It is observed that such configurations tend to concentrate at the interface. Among systems containing [DCA]^−^, [PF_6_]^−^, and [TfO]^−^ additives, the number density of such configurations is higher than that of the neat EG-KOH system throughout the liquid slab. However, for [EMIM]­[NTf_2_], the number density is lower than the neat EG-KOH absorbent at the interface but increases slightly within the bulk.

**Figure 8:**
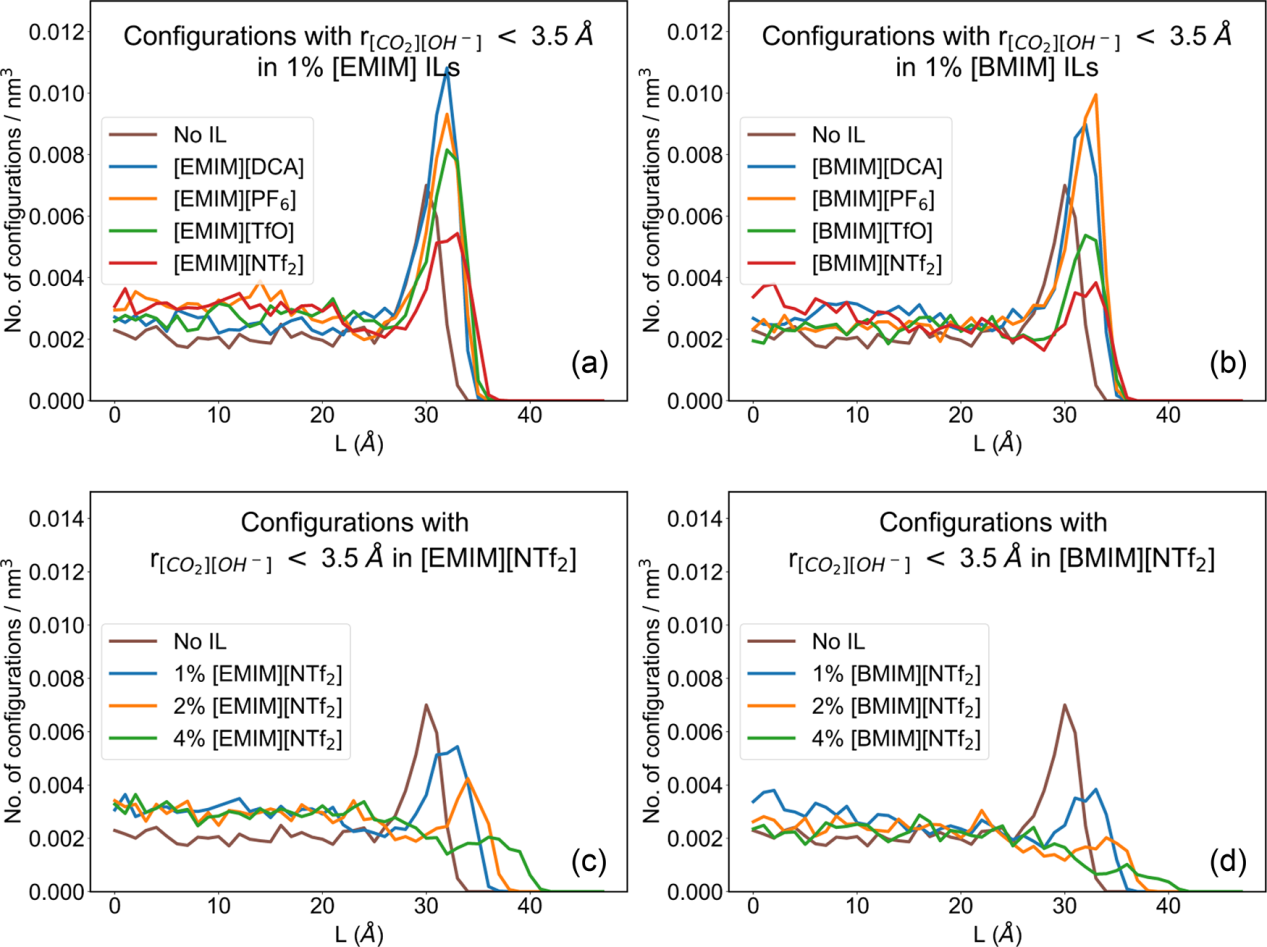
Distribution of configurations with *r*_[CO_2_][OH^–^]_ < 3.5 Å along direction normal to the gas–liquid interface in (a) 1% [EMIM] ILs and (b) 1% [BMIM]^+^ ILs (c) [EMIM]­[NTf_2_] systems (d) [BMIM]­[NTf_2_] systems at various concentrations.

Similarly, Figure 8b compares the number density of the configurations where *r*_[CO_2_][OH^–^]_ < 3.5 Å along the L-coordinate in systems with 1% [BMIM]^+^ additives. In [BMIM]­[DCA] and [BMIM]­[PF_6_], the number density is generally higher than that of the neat EG-KOH system throughout the liquid. For systems with [BMIM]­[TfO] or [BMIM]­[NTf_2_], the bulk contains a slightly higher density of favorable [OH]^−^-CO_2_ configurations but lower than the neat EG-KOH system at the interface.

Figure 8c,8d illustrates the effect of concentration on the distribution of favorable [OH]^−^-CO_2_ configurations along the L-coordinate for systems containing [EMIM]­[NTf_2_] and [BMIM]­[NTf_2_], respectively. It is evident that with increasing [NTf_2_]^−^ based IL concentration, the number density at the interface progressively decreases, albeit remaining slightly better than that for the EG-KOH system within the bulk. In fact, at 4% IL concentration, the concentration profiles reverse, and the interface becomes less favorable for the [OH]^−^-CO_2_ interactions relative to the bulk. This implies that a molecule of dissolved CO_2_ needs to diffuse deeper into the bulk liquid before it can favorably interact with an [OH]^−^ ion, thereby slowing the reaction rate. These findings are crucial and indicate that excessive additives can potentially degrade the CO_2_ capture performance.

### Experimental Observations

MD simulations strongly indicate that the addition of ILs can alter the dissolved CO_2_ concentration near the interface and the localization of [OH]^−^ around CO_2_. These being critical intermediate steps toward reactive CO_2_ capture, it is expected that IL additives also impact the overall reactive CO_2_ uptake. However, because of the limitations of using a nonreactive force field, this could not be confirmed computationally. In contrast, the highly reactive nature of the mixture makes the experimental determination of the additive’s impact on the individual physicochemical properties challenging. The assessment of the differences in the localization of the reactants at subnanometer length scales is also challenging. Thus, the experimental verification of the exact results obtained through MD simulations is beyond the scope of this work. However, the consumption of [OH]^−^ ions is the net result of all thermodynamic, transport, and chemical phenomena and is also significant from the perspective of process design. Hence, we measure the rate of [OH]^−^ conversion as a metric to qualitatively assess the impact of IL additives on the overall CO_2_ capture process.

Figure 9a depicts the differences in the experimentally measured [OH]^−^ conversion rates across systems containing [BMIM]­[DCA] and [BMIM]­[NTf_2_], each at a 1% molar concentration. It is evident that while the addition of ILs can accelerate the [OH]^−^ conversion rates, the choice of anion is important in determining the magnitude of the impact. The faster conversion in mixture with [BMIM]­[DCA] is in alignment with the results from the MD simulations, which suggested better localization of [OH]^−^ and CO_2_ near the interface of [DCA] ILs compared to the corresponding [NTf_2_] based systems. Figure 9b compares the experimental performance of systems containing [EMIM]­[NTf_2_] and [BMIM]­[NTf_2_], at an approximately 2% concentration. It can be observed that [EMIM]­[NTf_2_] performs better than [BMIM]­[NTf_2_], highlighting the effect of the choice of the cation on the overall performance. Figure 9c,d represents [OH]^−^ conversion as a function of [EMIM]­[NTf_2_] and [BMIM]­[NTf_2_] concentrations. The peak performance is observed at 2% [EMIM]­[NTf_2_] and 1% [BMIM]­[NTf_2_], respectively. These results reaffirm the effect of IL concentration on the overall performance and also suggest that optimum additive concentration can vary across ILs.

**Figure 9:**
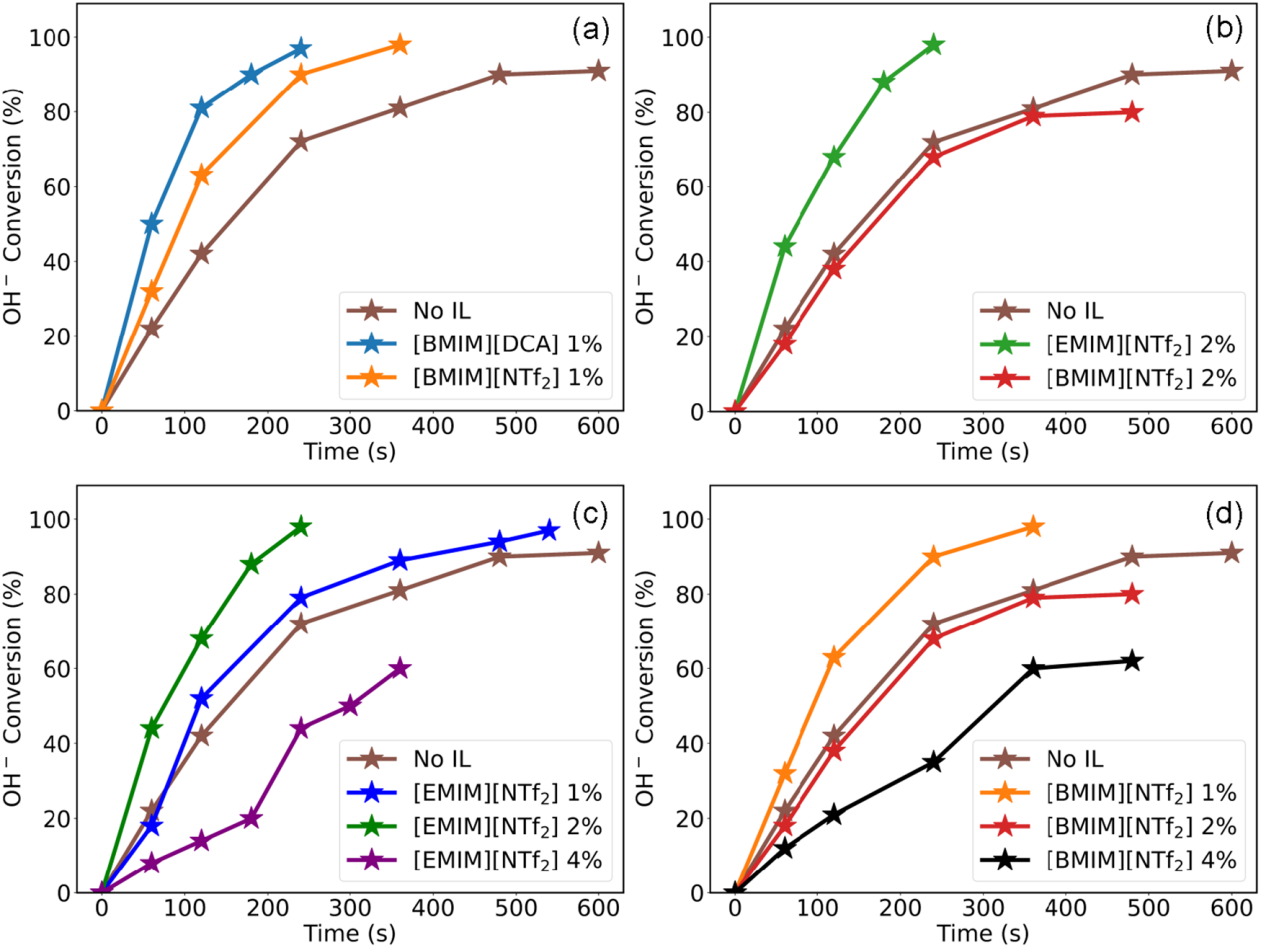
Experimental measurement of [OH]^−^ conversion as a function of time in systems with (a) 1% [BMIM] ILs, (b) 2% [NTf_2_] ILs, (c) [EMIM]­[NTf_2_] ILs, and (d) [BMIM]­[NTf_2_] ILs.

### Conclusions

MD simulations demonstrated that the cations and anions of ILs can undergo heterogeneous redistribution at the gas–liquid interface when added to a mixture of EG and KOH at low molar concentrations. The trends in the distribution of cations and anions at the interface are governed by the functional groups present on the ions. Specifically, imidazolium cations with longer alkyl chain substituents are solvophobic and migrate to the interface. Similarly, anions such as [NTf_2_]^−^ and [TfO]^−^ are likely to migrate to the interface, while anions such as [DCA]^−^ are solvophilic and are dispersed throughout the liquid. These differences in the distribution of ions impact the local thermophysical and chemical properties near the gas–liquid interface.

Next, we investigated the impact of IL additives on the surface tension and physical CO_2_ solubility of the mixtures. A 20% reduction in the surface tension values can be achieved at 4% IL concentration, while the *k*_HCO_2__ values are reduced by approximately 20% at 1% IL additive concentration. The dissolution and effusion behavior of CO_2_ was examined by monitoring the number of CO_2_ molecules dissolved in the liquid. At 1% IL concentration, the average number of dissolved CO_2_ molecules was improved by as much as 30% due to IL additives, particularly [BMIM]­[NTf_2_]. At 4%, the number increases further to about 50% higher than that of a mixture without an IL additive. The distribution of dissolved CO_2_ along the direction perpendicular to the IL interface indicates that nearly half of the dissolved CO_2_ lies close to the interface. Although this is likely to be influenced by the surface area/volume ratio of the system, it clearly establishes the importance of the gas–liquid interface toward reactive CO_2_ capture.

The use of a nonreactive force field limits us to the use of physical interactions between [OH]^−^ and CO_2_ to infer trends in reaction rates. We analyzed the localization of [OH]^−^ and CO_2_ within 3.5 Å of each other. The addition of ILs to the EG-KOH mixture helps bring [OH]^−^ and CO_2_ close and promotes the occurrence of such configurations, thereby improving the likelihood of a reaction. Examination of number density profiles suggests that this is due to the presence of bulky, asymmetric, solvophobic imidazolium cations in the solvation shell of [OH]^−^, which allow the CO_2_ molecule to get closer to [OH]^−^ more often as compared to the smaller and charge-dense [K]^+^ ion. Among IL additives with different anions, the distribution of favorable configurations with *r*_[CO_2_][OH^–^]_ < 3.5 Å occurs predominantly near the interface for the most solvophilic anions such as [DCA]^−^. The occurrence of these configurations near the interface implies a shorter diffusion path for a dissolved CO_2_ molecule before it meets an [OH]^−^ ion, potentially speeding up the overall conversion rate.

The experimental verification of the exact prereaction dynamics or subnanometer range localization characteristics suggested from MD results is challenging due to the reactive nature of the system and limitations of the current experimental setup. In contrast, the experimental measurement of [OH]^−^ conversion rates serves as a metric of the net effect of all physicochemical phenomena at play, and offers direct evidence of changes in the CO_2_ capture rates. Hence, we used experiments in tandem with nonreactive MD to investigate the impact of IL additives on the reactive CO_2_ capture process. Thus, MD simulations capture changes in the local physicochemical properties near the gas–liquid interface, while experimental results confirm the influence of the cations, anions, and IL concentration on the [OH]^−^ conversion rates.

While these results are encouraging, we caution our readers that CO_2_ capture is an inherently complex process and ILs are versatile molecules. It is possible that the improvement to the reaction rates arises from a synergistic combination of several factors discussed in this work, or those beyond the scope of this work. For example, the heterogeneous redistribution of IL cations and anions can create new compounds locally. A combination of [K]^+^ with an IL anion, or [OH]^−^ pairing with an IL cation, forms species such as [K]­[DCA] and [EMIM]­[OH]. The reaction mechanisms and reaction energy barriers around these species are likely very different compared to the one in an EG-KOH mixture without the additives. Other effects, such as catalysis or alternative reaction pathways, are also possible, motivating further exploration. While it is beyond the scope of this work to exhaustively probe all possible mechanisms, we present the computational and experimental results to motivate future investigations of such systems. Taken together, the experimental and computational results are complementary and provide insightful details to aid design of IL additives for enhancing reactive CO_2_ capture.

## Supplementary Material



## Notes

Surface tension estimated using MD simulations; Average number of CO_2_ molecules in liquid (out of 100); distribution of cations and anions across the liquid slab with [EMIM] based IL additives; distribution of COMs of IL cations and anions along the L-coordinate; number density of (a) [OH]^−^and (b) [NTf_2_]^−^ around CO_2_ in bulk liquid (PDF)
