# Supplementary material for: Ionic Liquid-Enhanced Interfaces to Boost Reactive CO2 Capture
Source: J Phys Chem B. 2026 Feb 19;130(9):2605–18. doi: 10.1021/acs.jpcb.5c07692 (PMC12969271; doi:10.1021/acs.jpcb.5c07692)
Supplement: Supplementary file 1 [file jp5c07692_si_001.pdf]

Supporting Information for Ionic Liquid-Enhanced Interfaces to Boost Reactive CO_2 Capture

Amey S. Thorat,[†] Rohan Sartape,[‡] Rohit Chauhan,[‡] Rashmi Mishra,[†] Meenesh R. Singh,[‡] and Jindal K. Shah*,[†]

†*School of Chemical Engineering, Oklahoma State University, Stillwater, Oklahoma 74078, United States*

‡*Department of Chemical Engineering, University of Illinois Chicago, Chicago, Illinois 60608, United States*

E-mail: jindal.shah@okstate.edu

S1 Supplementary Data

S1.1 Surface Tension

Table S1: Surface tension estimated using MD simulations

System	IL Concentration (mol %)	Mean Surface Tension (dyne/cm)	Std. Dev (dyne/cm)
EG + KOH	(No Additive)	50.2	0.31
[EMIM][DCA]	1%	50.2	0.26
[EMIM][PF_6]	1%	48.3	0.23
[EMIM][TfO]	1%	49.1	0.14
[EMIM][NTf_2]	1%	47.5	0.32
[EMIM][NTf_2]	2%	44.5	0.47
[EMIM][NTf_2]	4%	41.9	0.47
[BMIM][DCA]	1%	50.2	0.46
[BMIM][PF_6]	1%	49.3	0.23
[BMIM][TfO]	1%	48.4	0.38
[BMIM][NTf_2]	1%	47.5	0.30
[BMIM][NTf_2]	2%	43.8	0.36
[BMIM][NTf_2]	4%	40.4	0.43

S1.2 CO_2 Solubility

Table S2: CO_2 Henry's constant $k_{H_{CO_2}}$ estimated using MD simulations for systems with 1% [BMIM] additives.

System	IL Concentration (mol %)	$k_{H_{CO_2}}$ (bar) (bar)
EG + KOH	0%	487
[BMIM][DCA]	1%	415
[BMIM][PF_6]	1%	408
[BMIM][TfO]	1%	416
[BMIM][NTf_2]	1%	402

Table S3: CO_2 Henry's constant $k_{H_{CO_2}}$ at varying [BMIM][NTf_2] concentrations

(mol %)	$k_{H_{CO_2}}$ (bar)
0%	487
1%	426
2%	379
4%	310
5%	284
10%	201
20%	126
30%	92
40%	73
50%	60
60%	51
70%	44
80%	39
90%	35
100%	32

S1.3 Dissolved CO_2

Table S4: Average number of CO_2 molecules in liquid (out of 100)

System	IL Concentration (mol %)	Avg. no. of dissolved CO_2	Std. Dev
EG + KOH	0%	31	6.7
[EMIM][DCA]	1%	35	6.7
[EMIM][PF_6]	1%	38	6.5
[EMIM][TfO]	1%	40	6.2
[EMIM][NTf_2]	1%	43	5.9
[EMIM][NTf_2]	2%	46	5.8
[EMIM][NTf_2]	4%	50	5.4
[BMIM][DCA]	1%	36	6.5
[BMIM][PF_6]	1%	36	6.3
[BMIM][TfO]	1%	40	6.3
[BMIM][NTf_2]	1%	45	6.2
[BMIM][NTf_2]	2%	46	5.7
[BMIM][NTf_2]	4%	49	5.8

S1.4 $[OH]^-$-CO_2 Interactions

Table S5: Average number of occurrences with $r_{[CO_2][OH^-]} < 3.5$ Å per 2500 snapshots

System	IL Concentration (mol %)	No. of occurrences	Std. Dev
EG + KOH	0%	1088	102
[BMIM][DCA]	1%	1508	84
[BMIM][PF_6]	1%	1442	22
[BMIM][TfO]	1%	1190	106
[BMIM][NTf_2]	1%	1250	76
[BMIM][NTf_2]	2%	1049	59
[BMIM][NTf_2]	4%	980	104
[EMIM][DCA]	1%	1505	131
[EMIM][PF_6]	1%	1598	105
[EMIM][TfO]	1%	1492	145
[EMIM][NTf_2]	1%	1464	154
[EMIM][NTf_2]	2%	1388	174
[EMIM][NTf_2]	4%	1388	67

S2 Supplementary Figures

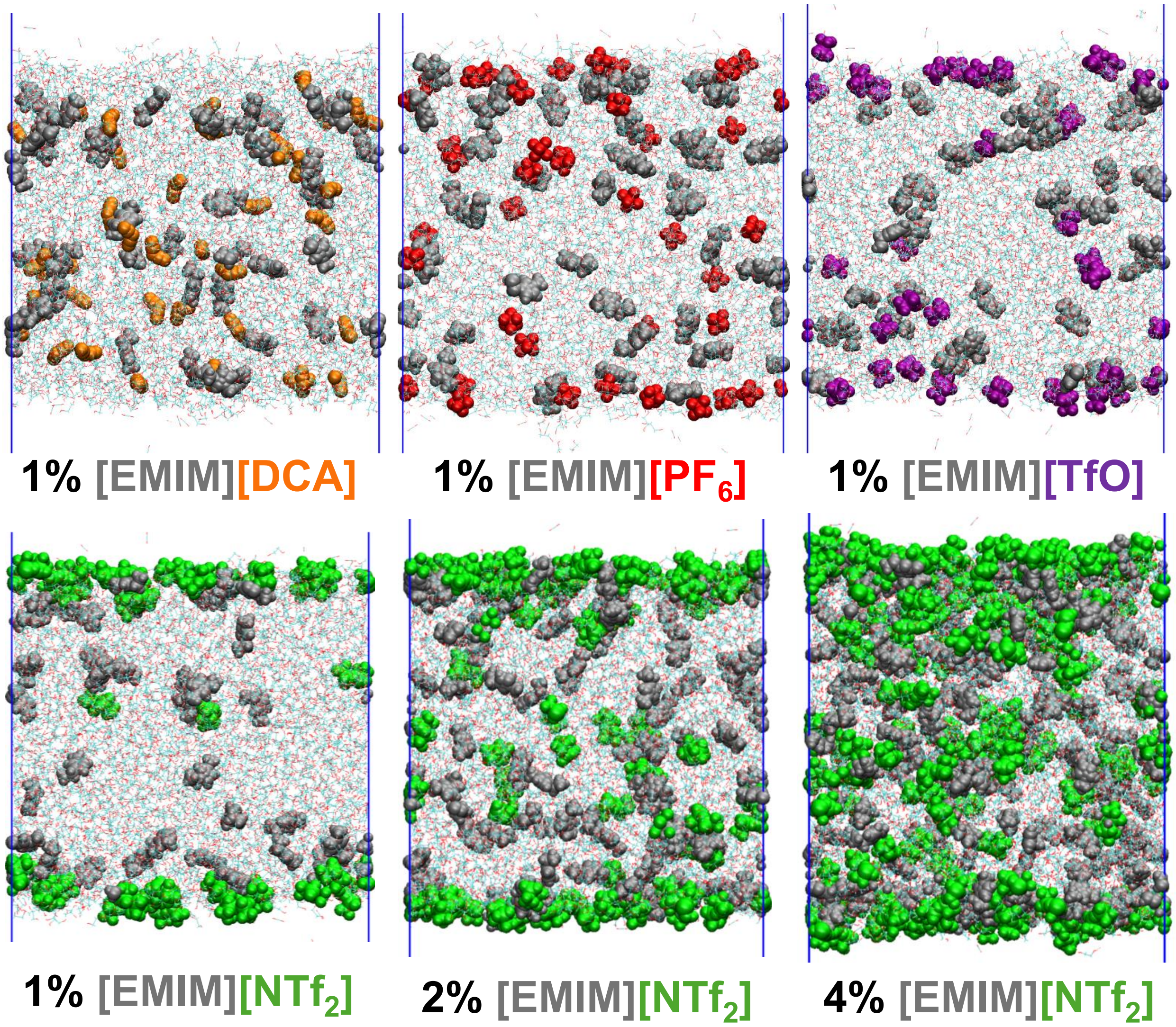

Figure S1: Distribution of cations and anions across the liquid slab with [EMIM] based IL additives.

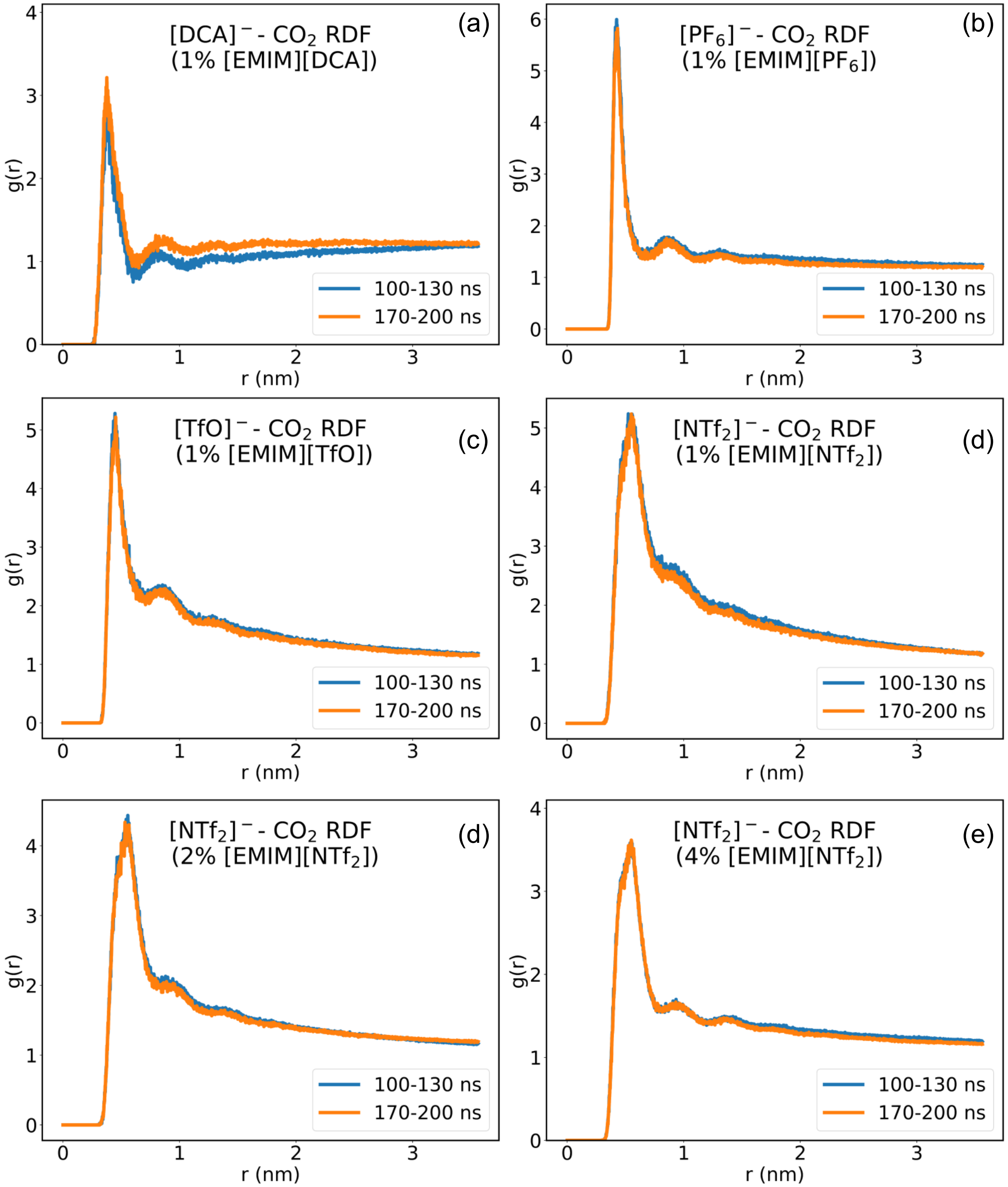

Figure S2: Anion-CO_2 COM RDFs calculated over the initial and final 30 ns of the production run trajectory in the liquid slab of systems containing $[EMIM]^+$ IL additives.

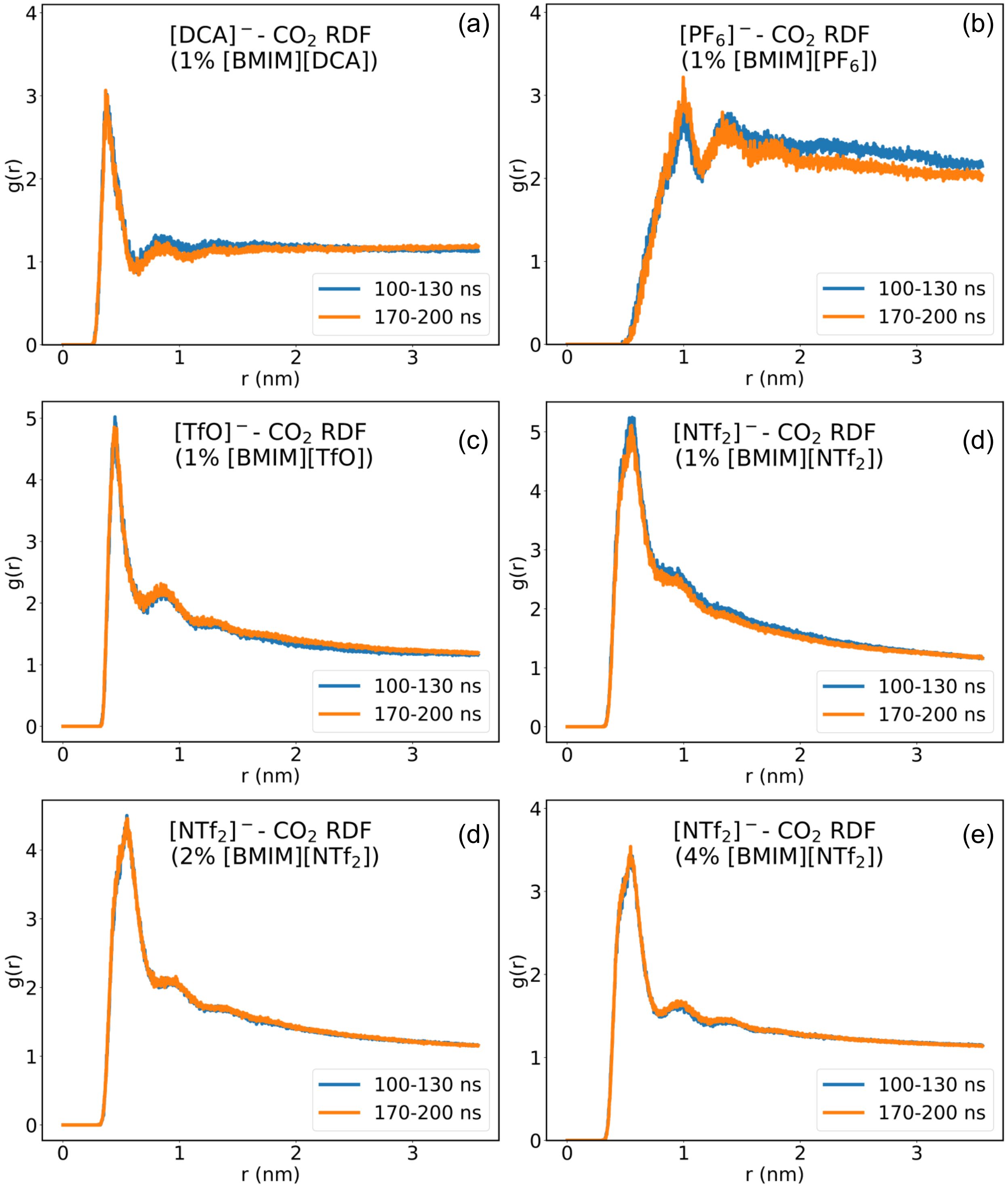

Figure S3: Anion-CO_2 COM RDFs calculated over the initial and final 30 ns of the production run trajectory in the liquid slab of systems containing $[BMIM]^+$ IL additives.

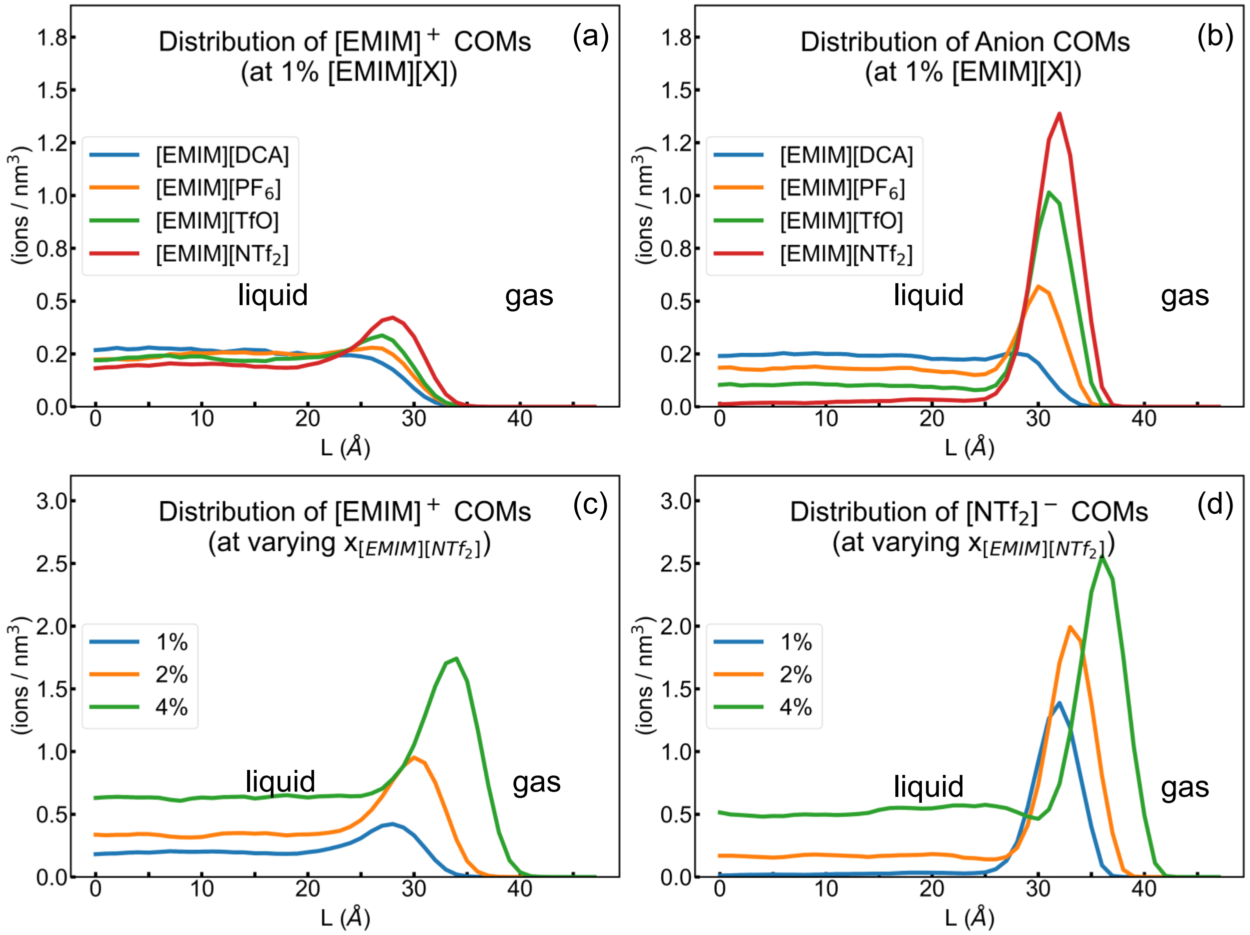

Figure S4: Distribution of COMs of IL cations and anions along the L-coordinate a) $[EMIM]^+$ COMs in 1% [EMIM] ILs b) Anions in 1% [EMIM] ILs c) $[EMIM]^+$ in [EMIM][NTf_2] and d) $[NTf_2]^-$ in [EMIM][NTf_2] systems.

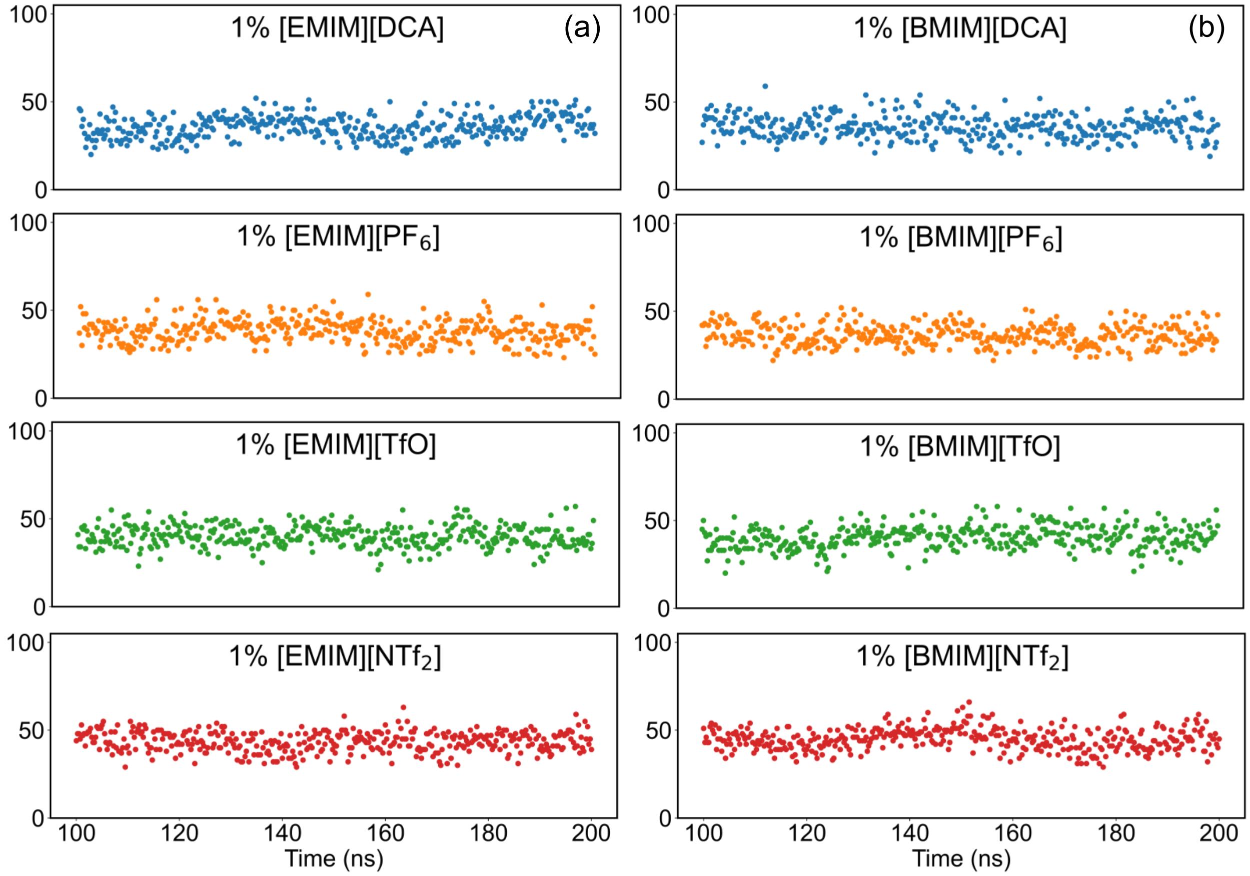

Figure S5: Number of dissolved CO_2 molecules (out of 100) evolving over time in systems with a) 1% [EMIM] IL additives and b) 1% [BMIM] IL additives.

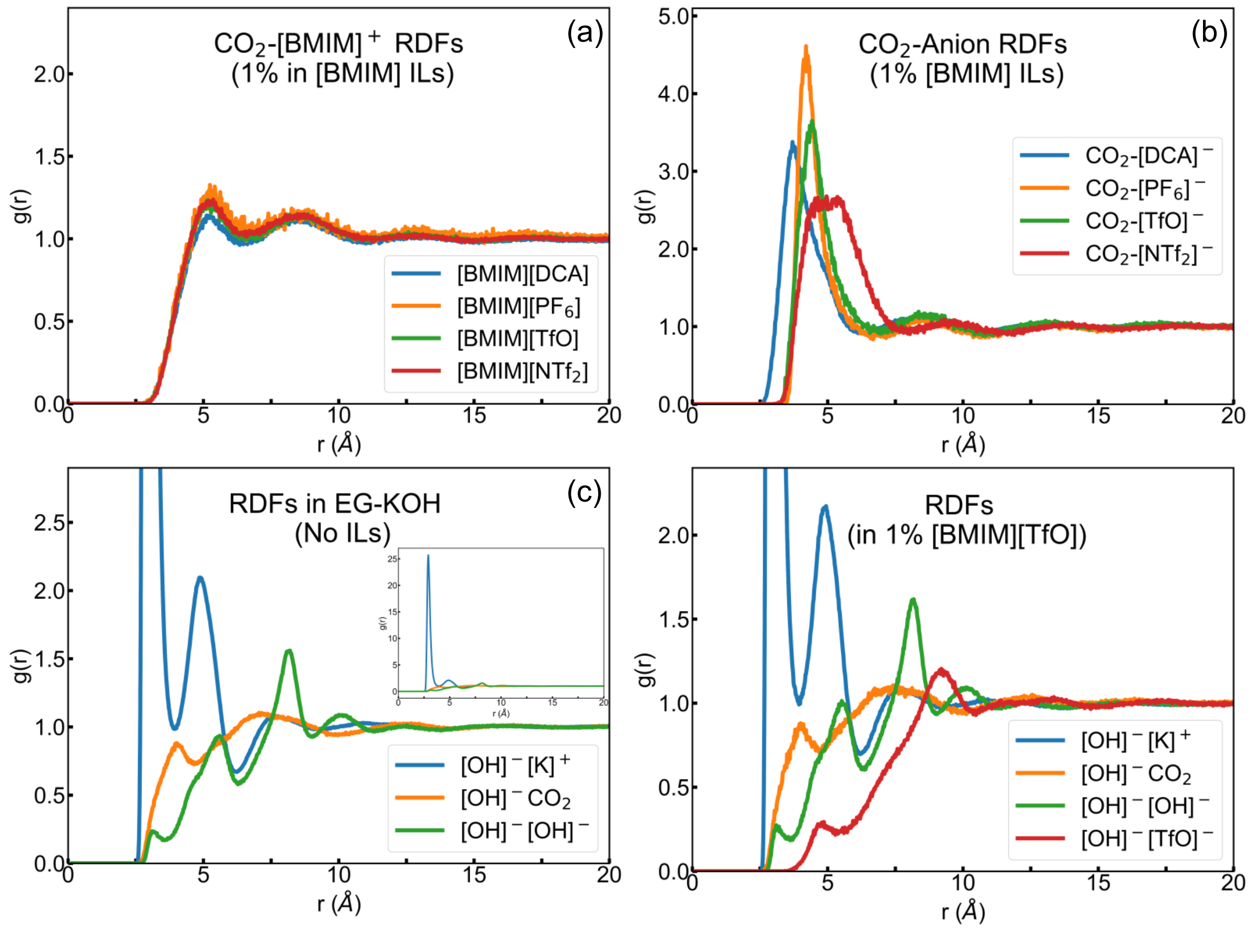

Figure S6: a) CO_2-[BMIM]$^+$ RDFs in systems with 1% [BMIM] IL additives b) CO_2-Anion RDFs in systems with 1% [BMIM] IL additives c) RDFs in EG-KOH system (with no ILs) d) RDFs with 1% [BMIM][TfO]

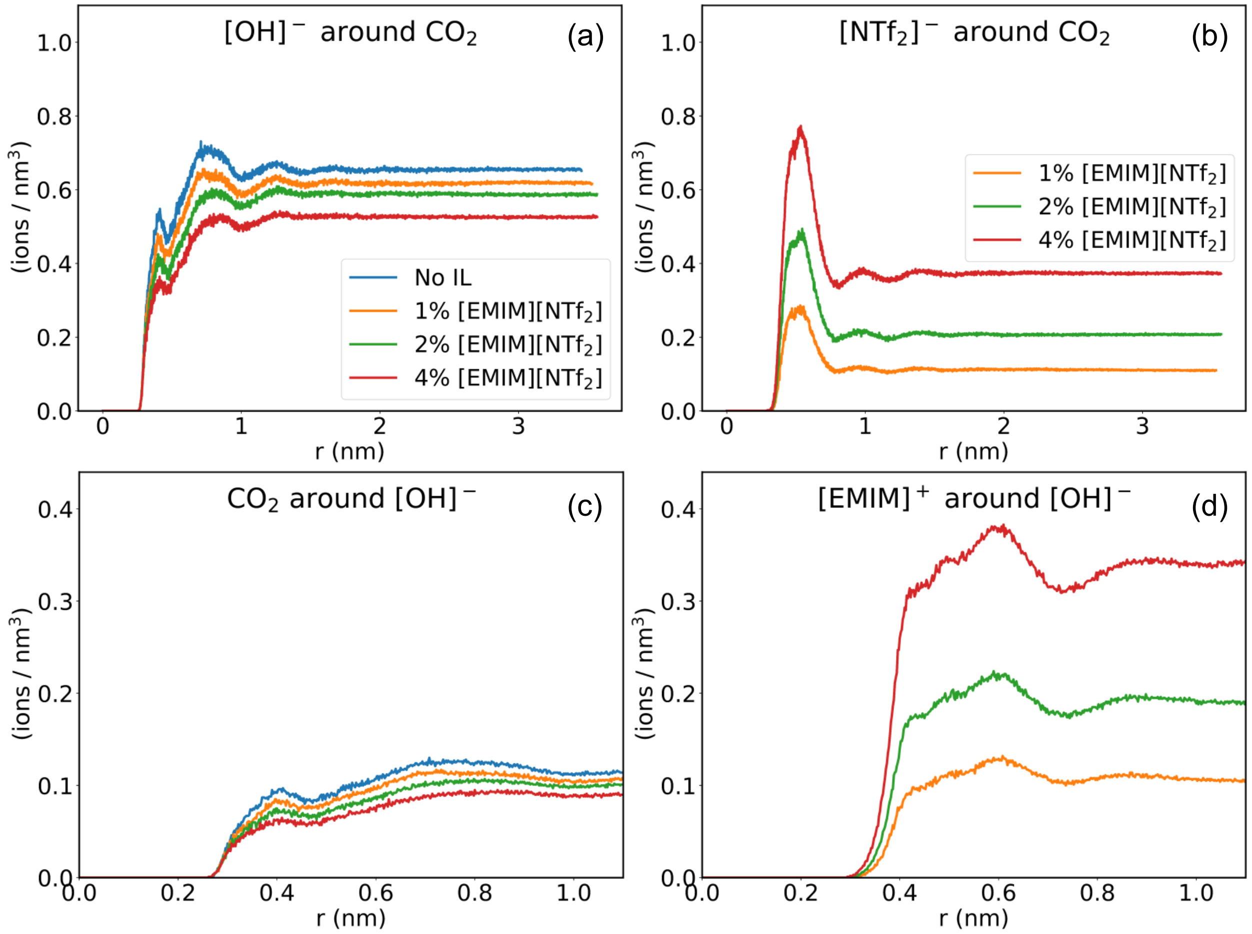

Figure S7: Number density of a) $[OH]^-$ and b) $[NTf_2]^-$ around CO_2 in bulk liquid. Number density of c) CO_2 and d) $[EMIM]^+$ around $[OH]^-$ in bulk liquid.

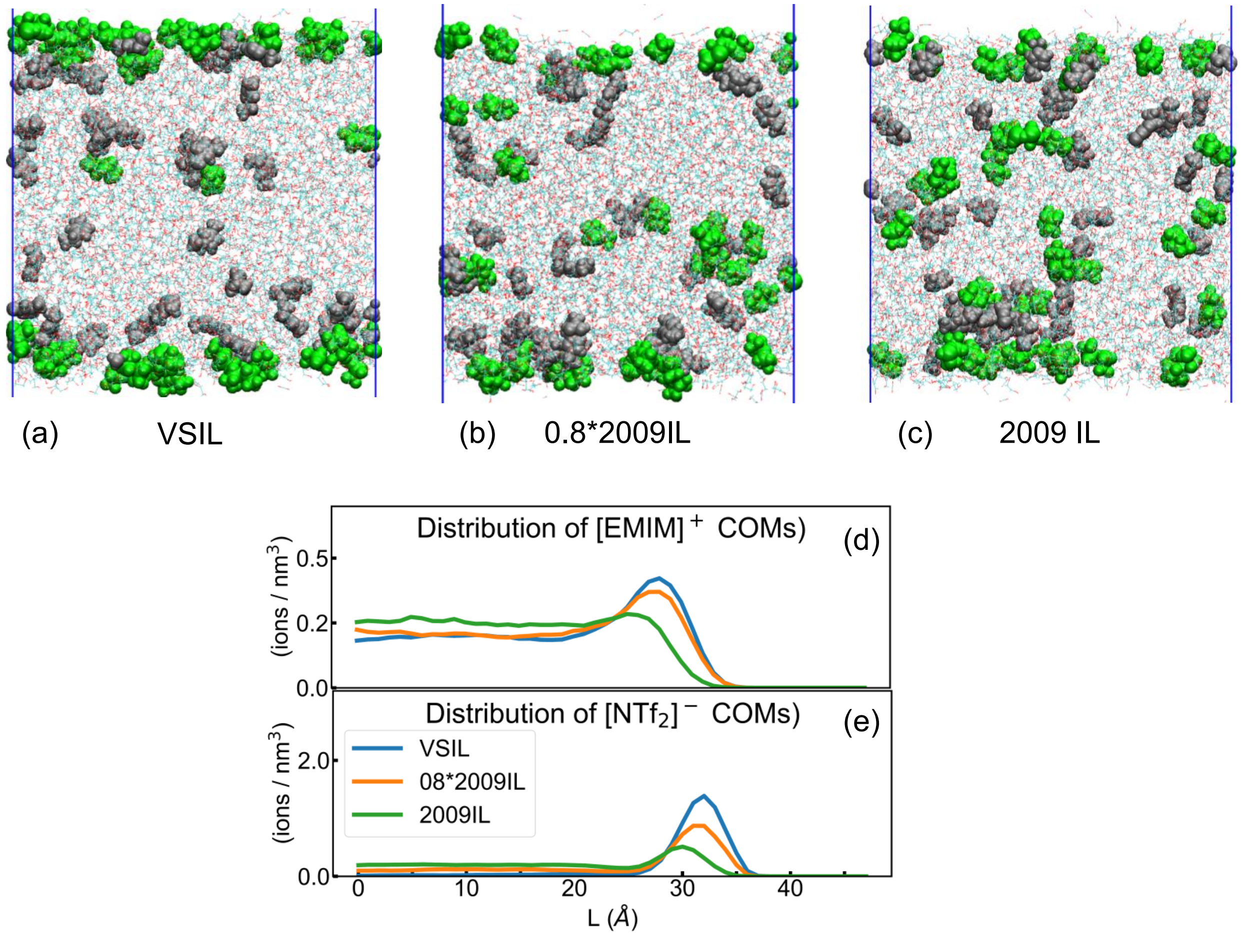

Figure S8: Snapshots showing distribution of $[EMIM]^+$ (grey) and $[NTf_2]^-$ (green) in systems with 1% $[EMIM][NTf_2]$ additive simulated using a) VSIL b 0.8*2009IL, and c) 2009IL force fields respectively. Effect of force field on the number density distribution of COMs of d) $[EMIM]^+$ and e) $[NTf_2]^-$ in systems containing 1% $[EMIM][NTf_2]$ additive.
